# Explainability-informed patient-disjoint evaluation of deep learning for plus disease in retinopathy of prematurity with an ophthalmologist reader study

**DOI:** 10.3389/fmed.2026.1960317

**Published:** 2026-09-30

**Authors:** Rouhollah Kian Ara, Jan Kubíček, Sumit Kaushik, Kristýna Maršolková, Juraj Timkovič

**Affiliations:** 1 Department of Cybernetics and Biomedical Engineering, VSB–Technical University of Ostrava, Ostrava, Czech Republic; 2 Clinic of Ophthalmology, University Hospital Ostrava, Ostrava, Czech Republic; 3 Department of Ophthalmology, Faculty of Medicine, University of Ostrava, Ostrava, Czech Republic

**Keywords:** deep learning, neonatal fundus imaging, ophthalmologist reader study, patient-disjoint evaluation, plus disease, retinopathy of prematurity, within-infant variability

## Abstract

**Background:**

Plus disease is a treatment-relevant sign of retinopathy of prematurity, but fundus images from the same infant may differ in image quality, posterior-pole coverage, and visible vascular severity. We evaluated deep-learning classification using a patient-disjoint design, within-infant variability, and an ophthalmologist reader study.

**Methods:**

The primary analysis included 732 Phoenix ICON fundus images from 38 infants, assigned exclusively to a 634-image development pool or a 98-image hold-out set from eight previously unseen infants. Five pretrained convolutional neural networks were evaluated at the image level. Hold-out predictions were grouped by infant and laterality to characterize descriptive within-infant variation. A historical secondary analysis used a random image-level partition without enforced patient separation and was considered exploratory. Two ophthalmologists independently graded 30 unique isolated images for inter-observer analysis; 10 were presented twice, resulting in 40 presentations for intra-observer repeatability.

**Results:**

DenseNet121 achieved an image-level ROC-AUC of 0.929; however, infant-equal-weighted image-level ROC-AUC was 0.905 and leave-one-infant-out ROC-AUC ranged from 0.838 to 0.989, demonstrating sensitivity to the small clustered hold-out cohort. At a development-defined threshold of 0.0184, sensitivity increased to 98.0%, while specificity decreased to 46.8%. Within-infant probability variability was observed across all five CNN architectures. Ophthalmologists 1 and 2 assessed 14/18 and 17/18 plus-reference images, respectively, as Healthy. Exact three-category inter-observer agreement was 26/30 (86.7%), and both reproduced the same severity grade for all 10 repeated images.

**Conclusions:**

Patient-disjoint evaluation revealed substantial uncertainty in image-level performance and variability among images from the same infant. Cluster-aware analyses showed dependence on the small hold-out cohort, while the development-defined operating point demonstrated a marked sensitivity–specificity trade-off. The reader study showed that an examination-derived reference label and the diagnostic evidence visible in an isolated image are not equivalent, despite high inter-observer agreement and complete categorical repeatability. Because examination identifiers were unavailable, within-infant analyses were interpreted descriptively and not as infant or examination-level diagnostic performance. Clinically useful systems should assess image quality, select informative views, and integrate evidence across multiple images and both eyes. External prospective multicentre validation is required before clinical implementation.

## 1 Introduction

Retinopathy of prematurity (ROP) is a vasoproliferative retinal disorder affecting preterm infants and remains an important cause of potentially preventable childhood visual impairment worldwide (1, 2). The International Classification of Retinopathy of Prematurity, Third Edition (ICROP3), defines plus disease by abnormal dilation and tortuosity of the posterior-pole retinal vessels, whereas pre-plus disease represents vascular abnormalities greater than normal but insufficient for definite plus disease (3, 4). Plus disease is a treatment-relevant marker of severe ROP and contributes directly to decisions regarding laser photocoagulation or intravitreal anti-vascular endothelial growth factor therapy (2, 4). Delayed recognition of severe ROP may permit progression to retinal detachment and irreversible visual impairment. Accurate and timely assessment is therefore essential, but diagnosis remains dependent on ophthalmologists with specific expertise in neonatal retinal examination, whose availability may be limited in regions with restricted neonatal and ophthalmic services (1, 2).

Clinical grading of plus disease is inherently challenging. Assessment is based primarily on the degree of venous dilation and arterial tortuosity in the posterior pole and therefore depends on adequate visualization of the optic disc and major vascular arcades. Diagnostic disagreement among experts has been reported, particularly along the continuous spectrum from normal vascular appearance through pre-plus to definite plus disease (5, 6). Neonatal fundus imaging may also be affected by infant eye movement, eyelid obstruction, variable camera angle, uneven illumination, defocus, and incomplete retinal coverage. Consequently, images acquired during the same examination may differ substantially in anatomical content, image quality, and diagnostic adequacy. In routine clinical practice, several images from both eyes are therefore commonly reviewed before an examination-level diagnosis is established. A diagnosis of plus disease based on the complete examination does not imply that every individual image independently displays definite posterior-pole vascular abnormalities. This distinction is consistent with previous work showing that ROP severity grading can change when clinicians assess serial rather than single retinal images, highlighting the influence of image context on plus-disease interpretation (7).

This distinction has important implications for automated classification. When individual images inherit a reference label derived from the complete clinical examination, the resulting labels represent examination-derived weak supervision rather than independent image-level annotations of visible plus-disease severity. A model may therefore be evaluated on its ability to predict the examination-derived class from one image, even when the image does not contain all features required for an ophthalmologist to make a definite isolated-image diagnosis. Transparent reporting of the reference standard and prediction unit is consequently essential for clinically meaningful interpretation.

Automated plus-disease assessment has historically followed two broad approaches. Earlier systems quantified hand-engineered vascular biomarkers, including vessel tortuosity and dilation. Longitudinal ROPtool analyses demonstrated that these characteristics change with disease progression and treatment (8). More recent feature-based systems have combined quantified vascular measurements with conventional machine-learning classifiers (9). These approaches provide relatively direct vascular interpretability but depend strongly on the accuracy of vessel segmentation and may exclude clinically relevant information outside the extracted vascular tree.

Deep-learning models can instead learn discriminative representations directly from retinal fundus photographs. Artificial-intelligence methods have increasingly been investigated across ophthalmology, including for ROP screening and plus-disease assessment (10). The i-ROP deep-learning system demonstrated strong performance for automated plus-disease classification within its development and test datasets (11). Other work has explored examination-level deep-learning approaches; Hu et al. developed a neural-network framework that aggregated information from multiple fundus images within an examination to recognize ROP presence and severity (12). Multidimensional platforms have extended automated analysis to image quality, ROP stage, haemorrhage, posterior-retinal localization, and pre-plus/plus disease classification (13). Nevertheless, performance achieved during internal development may not transfer directly to new institutions, patient populations, or imaging systems. For example, multicentre evaluation of ROP.AI showed lower discrimination and specificity than reported during its original development (14). These findings emphasize the need for transparent reporting of the clinical reference standard, prediction unit, data-partitioning strategy, operating threshold, and intended clinical role.

More broadly, recent benchmarking in fundus-image AI has emphasized evaluation across multiple model configurations and clinically relevant performance measures rather than reliance on a single accuracy estimate, highlighting the importance of robust evaluation when comparing retinal deep-learning systems (15).

Published ROP deep-learning studies differ in the level at which predictions and outcomes are reported, including the image, eye, examination, and patient levels. The general term “classification” does not specify either the prediction unit or the level at which data independence was enforced. This distinction is particularly important when several correlated images are available from the same infant. If images from one infant occur in both model-development and test partitions, performance may be overestimated because the test observations are not clinically independent. Conversely, a patient-disjoint split prevents direct infant overlap but does not by itself convert image-level predictions into patient- or examination-level diagnoses. Explicit reporting of both the partitioning unit and prediction unit is therefore necessary for valid interpretation and comparison (11, 13, 16).

A further methodological question is whether explicit representation of the retinal vasculature improves plus-disease classification. Vessel segmentation in neonatal fundus images is technically challenging because retinal vessels may have low contrast, while choroidal structures, illumination artefacts, and image noise may be incorrectly identified as vessels. Models trained primarily on adult retinal datasets may therefore transfer poorly to neonatal ROP imaging (17). Although vessel-derived representations can emphasize vascular anatomy, segmentation errors may omit fine vessels, fragment vascular branches, distort apparent vessel calibre, or introduce false vascular structures. Moreover, binary vessel overlays may suppress contextual information related to posterior-pole location, haemorrhage, pigmentation, illumination, focus, and field of view.

Model explainability is also relevant when automated predictions are proposed for clinical decision support. Gradient-weighted Class Activation Mapping (Grad-CAM) can identify image regions associated with a model prediction and can be used to assess whether activation overlaps with clinically relevant retinal anatomy (18). However, Grad-CAM does not establish causal feature use, diagnostic validity, or reasoning equivalent to that of an ophthalmologist. Such visualizations should therefore be interpreted as qualitative anatomical plausibility assessments and considered together with discrimination, probability reliability, error analysis, and clinician evaluation.

The present study addressed these methodological and clinical questions using two complementary image-level analytical strategies. The primary analysis used a patient-disjoint partition in which all images from the same infant were restricted to either the development pool or the hold-out set, thereby preventing direct infant overlap. Predictions and performance measures nevertheless remained image-level.

A secondary random image-level analysis, in which patient separation was not enforced, was retained as an exploratory supportive analysis to examine architecture behaviour, training-data sensitivity, probability reliability, model errors, Grad-CAM activation, and raw-fundus vs. vessel-overlay representations. Because complete infant-level provenance could not be reconstructed retrospectively, results from this historical dataset were not interpreted as evidence of independent patient-level generalization.

An ophthalmologist reader study further examined the relationship between isolated-image assessment and examination-derived clinical reference labels.

The objectives of the study were to:

1. quantify discrimination between plus-labelled and non-plus-labelled fundus images under a patient-disjoint partition, thereby evaluating image-level generalization to images from previously unseen infants;
2. characterize within-infant variability in image-level predicted plus-disease probabilities, including eye-specific distributions and ordered image-to-image variation, without interpreting these observations as infant- or examination-level diagnostic performance;
3. characterize model discrimination, probability reliability, error patterns, and sensitivity to training-data availability in a complementary random image-level analysis in which patient separation was not enforced;
4. determine whether explicit highlighting of the retinal vasculature using U-Net-derived vessel overlays improved plus-disease classification relative to the corresponding unmodified raw-fundus images;
5. evaluate how ophthalmologists graded isolated fundus images when the reference label was derived from the complete multi-image clinical examination, and assess intra-observer repeatability using repeated image presentations; and
6. qualitatively assess whether Grad-CAM activation patterns overlapped with clinically relevant retinal regions, while avoiding interpretation of such overlap as evidence of causal or ophthalmologist-equivalent model reasoning.

## 2 Materials and methods

### 2.1 Ethics approval

The retinal fundus images used in this retrospective study were acquired under a research project approved by the Ethics Committee of University Hospital Ostrava, Czech Republic (reference no. 544/2018), entitled “Software Application for the Assessment of Structural Retinal Changes in Prematurely Born Children.” The project received a favourable ethics opinion, and the clinical investigation was conducted at the Department of Ophthalmology, University Hospital Ostrava. All images used in the present analysis were de-identified before transfer to the research team and were processed in accordance with the approved protocol, institutional data-governance requirements, and the principles of the Declaration of Helsinki.

### 2.2 Dataset and clinical reference standard

All retinal fundus images were acquired using a Phoenix ICON wide-field retinal imaging system at a native resolution of 1240×1240 pixels. Multiple images from one or both eyes were available for some infants. Images sharing the same available infant identifier were treated as belonging to the same infant and were grouped accordingly for dataset partitioning.

Binary plus and non-plus reference labels were derived from the available clinical reference associated with each image set. These labels were assigned by one of two experienced ROP ophthalmologists (J.T. and K.M.) after review of the complete set of available fundus images associated with the corresponding clinical case. Each infant was assessed by a single ophthalmologist, and the identity of the grader responsible for each individual case was not retained in the retrospective dataset. The classification dataset did not contain a separate pre-plus reference category or independent severity annotations for each individual photograph. Accordingly, the reference standard was derived from the clinical image set rather than from independent grading of each photograph.

Individual photographs inherited the plus or non-plus reference label of the clinical image set from which they originated. Therefore, a plus-labelled photograph indicates that it originated from a case classified clinically as plus disease, but does not necessarily imply that definite plus-disease features were independently visible in that isolated image. The resulting labels should therefore be regarded as weak image-level supervision derived from the clinical reference rather than as independent image-level annotations of visible plus-disease severity. No separate multi-grader consensus or adjudication procedure was used for the original binary reference labels.

Clinical and acquisition metadata were available to varying degrees in the parent Phoenix ICON archive. The source records included demographic and clinical information such as gender, gestational age at birth, birth weight, age at successive imaging series, and longitudinal clinical diagnosis. However, a verified correspondence between these original clinical metadata and all images included in the derived plus/non-plus classification cohort was not available. Consequently, these variables could not be assigned reliably across the complete classification dataset for development-vs.-hold-out comparison or stratified analysis and were not retrospectively inferred or imputed.

ROP stage was present in some source clinical diagnosis entries, but was not available as a standardized image-level variable across the classification cohort. Detailed ROP zone, treatment history, eye-specific clinical diagnosis, verified visit identifiers, acquisition date, and operator information were likewise not available as consistently linkable variables for the complete classification dataset. Pre-plus status was not encoded as a separate reference category in the classification metadata and was therefore not retrospectively assigned.

#### 2.2.1 Primary patient-disjoint image-level dataset

The primary dataset constituted the main analysis of this study and comprised 732 retinal fundus images from 38 infants. Multiple images were available for most infants, including images from the right and left eyes when available.

A patient-disjoint split was performed using a fixed random seed of 42. The development pool contained 634 images from 30 infants, including 331 plus-labelled and 303 non-plus-labelled images. The independent patient-disjoint hold-out set contained 98 images from eight previously unseen infants, including 51 plus-labelled and 47 non-plus-labelled images. The complete dataset partition is reported in Supplementary Table S1.

The data partition was performed at the infant level, whereas predictions and performance measures were calculated at the image level. All images associated with the same available infant identifier were assigned exclusively to one partition, ensuring that no infant identifier appeared in both the development and hold-out sets. The 98 hold-out observations therefore represented 98 image observations from eight infants rather than 98 independent patient-level cases.

Within the hold-out set, images associated with three infant identifiers carried plus reference labels and images associated with five infant identifiers carried non-plus reference labels. Individual hold-out infants contributed between 1 and 30 images. Because detailed clinical and acquisition covariates were not consistently available, clinical spectrum balance between the development and hold-out cohorts could not be assessed.

#### 2.2.2 Historical secondary image-level dataset

A separate historical secondary dataset comprised 834 retrospectively collected Phoenix ICON fundus images, including 471 plus-labelled and 363 non-plus-labelled images. Stratified random sampling was performed independently within each class, with approximately 10% of the images allocated to a held-out random image-level test set. A fixed random seed of 42 was used for reproducibility.

The resulting development pool contained 751 images, including 424 plus-labelled and 327 non-plus-labelled images, and the held-out image-level test set contained 83 images, including 47 plus-labelled and 36 non-plus-labelled images. Thus, 751 images represented only the development portion of this historical secondary dataset and not the total dataset used in the study.

The development pool was used for cross-validation, model training, model selection, threshold exploration, and training-fraction analyses. The 83-image test set was excluded from these procedures and was used only for final exploratory image-level evaluation.

The development pool comprised 39 labelled image collections: 15 plus-labelled collections containing 424 images and 24 non-plus-labelled collections containing 327 images. Reference labels were derived from the available examination-level clinical assessment, and individual images inherited the corresponding examination-derived label. Consequently, an individual image from a plus-labelled examination did not necessarily show definite plus-disease features when viewed in isolation.

Images were sampled at the image level without using infant identity as a grouping constraint. Patient separation was therefore not enforced. Complete infant-level identifiers and split provenance were not available for all historical images at the time of the present revision, and the degree of infant overlap across development, validation, and test partitions could not be reconstructed retrospectively.

Within the 751-image development pool, five-fold cross-validation was stratified by image label rather than grouped by infant. Images from the same infant could therefore have occurred in different training and validation folds.

The historical secondary analyses were retained to address complementary methodological questions concerning image-level model behaviour, architecture comparison, training-data sensitivity, probability reliability, qualitative visualization, and alternative input representations. Because patient separation was not enforced and complete infant-level provenance could not be reconstructed retrospectively, these analyses may be optimistic because of within-infant dependence. They are therefore interpreted only as exploratory supportive image-level analyses, not as independent patient-level validation, and are not compared with the primary patient-disjoint analysis as equivalent evidence of generalization.

### 2.3 Illustrative multi-image case analysis

To illustrate the relationship between examination-level diagnosis, single-image interpretation, and AI prediction, we performed a descriptive case analysis of one infant with a clinical reference diagnosis of plus disease. The examination contained multiple fundus images from both eyes acquired during the same clinical visit. Nine images were available from the left eye and ten from the right eye. Twelve representative images were selected for visualization to demonstrate within-examination variation in field of view, illumination, focus, retinal coverage, and visibility of the optic disc and posterior-pole vessels.

One image from this examination had also been included in the independent ophthalmologist questionnaire. Each ophthalmologist assessed that image without access to the remaining images from the examination and assigned a severity category and confidence score. The isolated reader-study image was compared descriptively with the examination-derived clinical reference and with the remaining available images from the same clinical visit. No infant- or examination-level model performance was estimated from this illustrative case.

This case analysis was descriptive and was not used to estimate diagnostic accuracy or to perform statistical hypothesis testing. Grad-CAM maps were reviewed qualitatively to assess whether model activation overlapped with clinically relevant retinal vascular regions. The activation maps were interpreted as anatomical plausibility checks and not as evidence of causal or ophthalmologist-equivalent reasoning.

### 2.4 Study design and analytical hierarchy

This retrospective study evaluated binary classification of plus-labelled vs. non-plus-labelled fundus images using two partitioning strategies. The primary analysis used a patient-disjoint split, ensuring that all images from the same infant were assigned to only one partition. Although the split was performed at the infant level, model predictions and performance measures were calculated for individual images. The secondary analysis used a random image-level split without enforced patient separation and was considered supportive and exploratory. The ophthalmologist reader study evaluated isolated-image grading and should not be interpreted as a measurement of full examination-level diagnostic accuracy. The analytical components, prediction units, datasets, and intended interpretations are summarized in Table 1.

**Table 1:** Analytical components, prediction units, and interpretation.

Component	Analytical unit	Data	Interpretation
Primary patient-disjoint image-level analysis	Individual fundus image	98 images from eight infants; 51 plus-labelled and 47 non-plus-labelled	All images from each infant were restricted to one partition. Performance was calculated at the image level and represents generalization to images from previously unseen infants.
Exploratory infant-grouped aggregation	One aggregated output per infant	The same 98 images grouped into eight infants	Four post hoc aggregation rules were compared descriptively. Results do not represent validated infant-level diagnostic performance.
Image-to-image variability analysis	Adjacent available image pair within one infant–laterality group	84 image pairs	Assessed changes in predicted probability between adjacent available images from the same infant and laterality group. Image order was used only for descriptive comparison and was not interpreted as verified acquisition timing or biological progression.
Secondary random image-level analysis	Individual fundus image	83 held-out images; 47 plus-labelled and 36 non-plus-labelled	Images were sampled randomly without enforced patient separation. Results represent exploratory image-level performance within the available collection.
Single-image ophthalmologist assessment	Isolated image presentation	30 unique images and 10 repeated presentations	Evaluated isolated-image grading, inter-observer agreement, and intra-observer repeatability. It was not a test of complete-examination diagnostic accuracy.
Illustrative multi-image case	One clinical examination	Images from both eyes of one clinically diagnosed plus-disease infant	Provided descriptive context for discordance among the clinical reference, isolated-image assessments, and model outputs. No statistical inference was performed.

### 2.5 Model architectures, training, and inference

All retinal fundus images were preprocessed using a custom automated cropping algorithm designed to remove black background borders introduced during image acquisition. For each image, a binary foreground mask was generated by thresholding the mean pixel intensity across the red, green, and blue channels at 10. The bounding box of all non-black pixels was identified, and a square region was extracted by centring the crop on the bounding box and adding a 10-pixel margin. The cropped images were then resized to 224×224 pixels to match the input dimensions of the convolutional neural network backbones. Pixel values were normalized using the ImageNet mean values (0.485,0.456,0.406) and standard deviations (0.229,0.224,0.225). Automated foreground cropping reduced variation attributable to the surrounding black image border; however, no dedicated shortcut-control experiment was performed. Differences in illumination, field of view, focus, retinal coverage, acquisition angle, or other image-acquisition characteristics could therefore remain available to the classifier. Formal image-quality, gradability, and posterior-pole adequacy annotations were not available for the retrospective image collection. No images were therefore excluded post hoc on the basis of a subjective or retrospectively defined quality score beyond the predefined image eligibility and preprocessing procedures. During training, the following stochastic data-augmentation procedures were applied to increase image variability and reduce overfitting:

- random horizontal flipping with a probability of 0.5;
- random rotation within $\pm 10^{\circ }$;
- colour jitter with brightness, contrast, and saturation factors of 0.1 and a hue factor of 0.02.

No augmentation was applied during validation or final testing.

#### 2.5.1 Shared model architecture and training protocol

Five convolutional neural network architectures pretrained on ImageNet were fine-tuned for binary classification of plus disease: EfficientNet-B0 (19), DenseNet121 (20), ResNet50 and ResNet-18 (21), and MobileNetV3 Large (22).

The architecture panel was selected to provide a controlled comparison across several established ImageNet-pretrained CNN families rather than to identify a state-of-the-art backbone. ResNet-18 and ResNet50 represent residual networks with different model capacities, DenseNet121 provides dense feature connectivity, EfficientNet-B0 uses compound scaling, and MobileNetV3 Large represents a computationally lightweight architecture. The study was therefore designed to assess whether the principal findings were consistent across different established CNN families under the same data-partitioning and training framework, rather than to provide an exhaustive benchmark of contemporary vision architectures. For each architecture, the original classification head was replaced with a fully connected linear layer producing two output logits corresponding to the non-plus and plus classes. Softmax activation was applied during inference to obtain the predicted plus-disease probability.

The same model architectures, ImageNet initialization, preprocessing, data-augmentation procedures, optimization settings, number of training epochs, batch size, loss function, checkpoint-selection criterion, and fold-ensemble inference procedure were used in both the primary patient-disjoint analysis and the secondary random image-level analysis. The two analyses differed in dataset composition and data-partitioning strategy. The primary analysis used patient-disjoint partitioning with infant-grouped cross-validation, whereas the historical secondary analysis used random image-level partitioning and image-label-stratified cross-validation without infant grouping.

Each fold-specific model was trained for 200 epochs using the AdamW optimizer with a fixed learning rate of $1\times 10^{-4}$, a batch size of 16, and cross-entropy loss. Model checkpoints were saved after each epoch, and the checkpoint with the highest validation accuracy was retained for the corresponding fold. Automatic mixed-precision training was disabled. A fixed random seed of 42 was applied to Python’s random module, NumPy, and PyTorch to improve reproducibility of data partitioning, model initialization, and stochastic augmentation.

#### 2.5.2 Primary patient-disjoint model development

For the primary analysis, model development was performed within the 634-image development pool containing images from 30 infants. Five-fold subject-wise stratified group cross-validation was used, with infant identity serving as the grouping variable. All images associated with the same infant were assigned exclusively to either the training or validation subset within each fold. Consequently, no infant contributed images to both subsets in the same cross-validation iteration.

The independent 98-image hold-out set from eight infants was excluded from cross-validation, model fitting, validation, checkpoint selection, and threshold selection. For final evaluation, the five retained fold-specific models were applied to every hold-out image. Their predicted plus-disease probabilities were averaged to obtain one ensemble probability for each image. A threshold of 0.5 was retained as the reproducible reference operating point for the principal threshold-dependent analysis. An additional sensitivity-oriented operating point was selected exclusively from development data as described in Section 2.8.

Although data partitioning and cross-validation were performed at the infant level, the model input, prediction unit, and reported performance measures remained image-level.

#### 2.5.3 Secondary random image-level model development

For the secondary analysis, five-fold stratified cross-validation was performed within the 751-image development pool. The folds were stratified according to the binary image label, but infant identity was not used as a grouping constraint. Images from the same infant could therefore occur in different training and validation folds.

In each cross-validation iteration, four folds were used for training and one fold was used for validation. This procedure was repeated until every development image had been used once for validation. The fold-specific validation probabilities were concatenated to generate out-of-fold predictions for the complete development pool.

The held-out 83-image random image-level test set was excluded from model training, cross-validation, validation, and checkpoint selection. For final evaluation, the five retained fold-specific models were applied to each test image, and their predicted plus-disease probabilities were averaged to produce one ensemble probability per image. The fixed threshold of 0.5 was used for the principal threshold-dependent analysis.

Because infant identity was not used during data partitioning, the secondary analysis represents exploratory image-level performance and does not provide independent patient-level generalization.

### 2.6 Raw-fundus vs. vessel-overlay experiment

An image-level experiment compared VGG16 classifiers trained using two input representations: (1) cropped raw fundus images and (2) vessel-overlay images generated from the same fundus photographs. The vessel-overlay representation was produced using an in-house retinal vessel-segmentation model, as described below.

#### 2.6.1 Retinal vessel-segmentation model

Retinal vessel masks were generated using a five-level U-Net architecture (UNet2) based on the encoder–decoder design with skip connections (23). The encoder contained feature dimensions of 32, 64, 128, 256, and 512 channels, followed by a 1024-channel bottleneck. The decoder symmetrically reduced the feature dimensions through 512, 256, 128, 64, and 32 channels. Encoder features were transferred to the corresponding decoder stages through skip connections, and bilinear upsampling was used to restore spatial resolution.

The segmentation model was developed using expert-annotated Phoenix ICON fundus images. Owing to the native image resolution, training was performed using randomly sampled 640×640-pixel patches. Vessel-aware patch sampling was applied to increase the representation of foreground vessel pixels during training.

The network was optimized using the Adam optimizer with an initial learning rate of $1\times 10^{-4}$ and a batch size of 4 for a maximum of 1,000 epochs. The loss function combined binary cross-entropy and Dice losses, as defined in Equation 1:

$$L_{\mathrm{seg}}=0.3L_{\mathrm{BCE}}+0.7L_{\mathrm{Dice}}.$$ (1)

The loss was calculated only within the retinal region-of-interest mask. A ReduceLROnPlateau scheduler reduced the learning rate by a factor of 0.1 after 40 epochs without improvement in validation loss. The checkpoint with the highest validation Dice score was retained.

To reduce variability between vessel annotations, two segmentation models with the same architecture were trained independently using annotations provided by two ophthalmologists. During inference, their pixel-wise posterior probability maps were averaged, as defined in Equation 2:

$$P_{\mathrm{ensemble}}(x)=\frac{P_{\text{Ophthalmologist 1}}(x)+P_{\text{Ophthalmologist 2}}(x)}{2},$$ (2)

where P(x) denotes the predicted probability that pixel x belongs to a retinal vessel. This probability-level ensemble was used to generate the final vessel representation.

Full-resolution images were segmented using sliding-window inference with 640×640-pixel tiles and 50% overlap. Predictions in overlapping regions were averaged, and the resulting probability map was thresholded at 0.5 to obtain the binary vessel mask. The segmentation-network weights were fixed during the subsequent classification experiment.

#### 2.6.2 Vessel-overlay construction and classification

For the vessel-overlay representation, the predicted binary vessels were overlaid in green on the corresponding raw fundus image, while non-vessel regions were dimmed. The raw-fundus and vessel-overlay pipelines were derived from the same source images and used the same data partitions, but differed in input representation and colour-augmentation procedures.

Separate VGG16 classifiers were trained using the raw-fundus and vessel-overlay inputs. Both classifiers used the same image-level development and test partitions. They were trained for 50 epochs using class-weighted cross-entropy loss, the AdamW optimizer, a cosine-annealing learning-rate schedule, a batch size of 16, and mixed-precision training. Colour jitter was omitted for the vessel-overlay model to avoid altering the appearance of the predicted vessel mask.

This experiment compared two complete input-processing pipelines. Any observed performance difference may reflect vessel-segmentation errors, loss of information caused by background dimming, or sensitivity to the artificial overlay distribution. It therefore does not isolate the biological contribution of retinal vessels from that of the surrounding retinal image.

### 2.7 Single-image ophthalmologist assessment

Two ophthalmologists independently reviewed 40 image presentations: 30 unique images and 10 repeated images. Each image was presented independently, without companion images from the same infant and without AI output. Readers graded vascular appearance as Healthy (0), Borderline/Pre-plus (1), or Plus (2), and reported confidence on a five-point scale from 1 (very uncertain) to 5 (very confident).

The purpose of this reader study was to determine whether an isolated fundus image provided sufficient visible vascular information for confident plus-disease grading when the source label had been established from a multi-image clinical visit. The 10 repeated images assessed whether reader decisions were internally reproducible.

### 2.8 Statistical analysis

Plus disease was the positive class. At threshold t, predictions were summarized by true positives (TP), true negatives (TN), false positives (FP), and false negatives (FN). The principal threshold-dependent metrics were calculated as defined in Equations 3–6.: ?>

$$\begin{array}{c} \mathrm{Accuracy}=\frac{TP+TN}{TP+TN+FP+FN},\\ \mathrm{Sensitivity}=\frac{TP}{TP+FN}, \end{array}$$ (3)

$$\mathrm{Specificity}=\frac{TN}{TN+FP},\;\mathrm{PPV}=\frac{TP}{TP+FP},$$ (4)

$$\mathrm{NPV}=\frac{TN}{TN+FN},\;F_{1}=\frac{2TP}{2TP+FP+FN}.$$ (5)

The Matthews correlation coefficient was calculated as:

$$\mathrm{MCC}=\frac{TP\times TN-FP\times FN}{\sqrt{\left(TP+FP)(TP+FN)(TN+FP)(TN+FN\right)}}.$$ (6)

ROC-AUC and PR-AUC were used as threshold-independent discrimination measures. The Brier score summarized squared probabilistic error, as defined in Equation 7:

$$\mathrm{Brier}=\frac{1}{n}\sum_{i=1}^{n}(p_{i}-y_{i}{)}^{2},$$ (7)

where $p_{i}$ is the predicted probability of plus disease and $y_{i}\in \{0,1\}$ is the reference label. The Brier score reflects both discrimination and calibration and was not interpreted as calibration alone (24, 25).

For descriptive analyses in which Youden’s index was reported, it was defined as shown in Equation 8:

J=Sensitivity+Specificity−1. (8)

Youden’s index was not used to select the development-defined high-sensitivity operating point reported in the primary patient-disjoint hold-out analysis.

#### 2.8.1 Development-defined high-sensitivity operating point

In addition to the fixed reference threshold of 0.5, a sensitivity-oriented operating point was evaluated for each architecture. Threshold selection was performed exclusively using out-of-fold predictions generated within the 634-image patient-disjoint development pool. A target development sensitivity of at least 90% was specified. Among all thresholds satisfying this criterion, the threshold yielding the highest development specificity was selected; ties were resolved by selecting the highest threshold.

The resulting threshold was fixed before evaluation on the independent 98-image patient-disjoint hold-out set. No hold-out predictions or labels were used for threshold selection, and no Youden-index or other test-set optimization was performed. The 90% sensitivity target was introduced as an additional sensitivity-oriented analysis and should not be interpreted as a prospectively validated clinical operating point.

Because model outputs were generated for individual images rather than complete examinations, the proportion of hold-out images classified as plus was reported as the image-level flagged fraction. This quantity was not interpreted as an examination-level referral rate.

Wilson 95% confidence intervals were calculated for selected proportions in the secondary image-level analyses. These intervals treated individual images as observations and therefore did not account for possible within-infant correlation.

For $\hat{\;p}=x/n$ and z=1.96, the Wilson interval was calculated as shown in Equation 9 as:

$$\frac{\hat{\;p}+\frac{z^{2}}{2n}\pm z\sqrt{\frac{\hat{\;p}(1-\hat{\;p})}{n}+\frac{z^{2}}{4n^{2}}}}{1+\frac{z^{2}}{n}}.$$ (9)

Because the primary hold-out set contained multiple images from only eight infants and the number of images contributed by each infant was unequal, three additional analyses were performed to evaluate the effect of within-infant clustering and cohort composition.

First, infant-equal-weighted image-level metrics were calculated. Each image from infant i was assigned a weight of $1/n_{i}$, where $n_{i}$ was the number of hold-out images contributed by that infant. Thus, the sum of the image weights for each infant was equal to one, preventing infants with more available images from contributing disproportionately to the calculated metrics. These weighted measures retained the individual image as the prediction unit and were therefore not interpreted as patient-level diagnostic performance.

Second, a leave-one-infant-out influence analysis was performed by successively excluding all images associated with one hold-out infant and recalculating performance using the remaining infants. The trained models and predicted probabilities were unchanged, and no model retraining or threshold optimization was performed. The fixed decision threshold of 0.5 was retained for all threshold-dependent measures.

Third, exploratory uncertainty intervals were estimated using a stratified infant-cluster bootstrap with 10,000 resamples. Infants rather than individual images were sampled with replacement. Plus- and non-plus-reference infant clusters were sampled separately to maintain representation of both classes, and all images belonging to each selected infant were retained together. The 2.5th and 97.5th percentiles of the bootstrap distributions were reported as exploratory 95% percentile intervals. Because the hold-out cohort contained only eight independent infants, including only three Plus-reference infants, these intervals were used to characterize cohort uncertainty and were not interpreted as precise population-level confidence intervals.

For the reader analysis, the 30 unique images were used for performance and inter-observer calculations, whereas the 10 repeated presentations were used only for intra-observer repeatability. Under the strict binary definition, only a definite Plus assessment was considered positive. Under the abnormality-sensitive definition, both Borderline/Pre-plus and Plus assessments were considered positive. Exact 95% confidence intervals for reader sensitivity and specificity proportions were calculated using the Clopper–Pearson method. Sensitivity, specificity, exact agreement, Cohen’s kappa, linear- and quadratic-weighted kappa, repeated-grade agreement, and confidence-score stability were reported.

The reader calculations used examination-derived clinical reference labels inherited by the individual images. They therefore measured agreement between isolated-image assessments and the available reference labels and should not be interpreted as estimates of ophthalmologist examination-level diagnostic accuracy.

### 2.9 Descriptive within-infant prediction variability

Image-level predictions from the primary patient-disjoint hold-out set were grouped by available infant identifier and eye laterality to characterize variation among images assigned to the same infant. Infant identity and eye laterality were available for each image. Images sharing the same infant identifier but different laterality were treated as separate infant–laterality groups.

The recorded image sequence index was used only to arrange the available images for descriptive comparison and was not interpreted as verified acquisition timing, elapsed clinical time, or biological disease progression. Complete examination dates and visit-level identifiers were not available; consequently, images assigned to the same infant could not be reliably partitioned into individual clinical examinations. No infant- or examination-level diagnostic performance was therefore estimated.

An exploratory infant-grouped analysis was performed using image-level predictions from the primary patient-disjoint hold-out set. DenseNet121 predictions were retained for detailed within-infant descriptive illustration, while the ordered within-infant variability analysis was additionally repeated across all five primary CNN architectures to assess whether the observed variability was architecture-specific.

The eight source infant identifiers were replaced with anonymized study identifiers P01–P08 before reporting. Images were grouped according to the following fixed order: P01, P02, P03, P04, P05, P06, P07, and P08. The analysis included 98 images from eight infants, comprising 43 left-eye and 55 right-eye images.

For each infant, the number of available images, the number of images from each eye, and the distribution of image-level predicted plus-disease probabilities were summarized. Descriptive measures included the mean, standard deviation, median, minimum, maximum, and the number of images with a predicted probability greater than or equal to the fixed image-level threshold of 0.5. Eye-specific mean probabilities were calculated when images from the corresponding eye were available.

Model outputs are described as predicted plus-disease probabilities rather than confidence scores. These values represent the softmax output of the classifier for individual images and should not be interpreted as calibrated measures of clinical diagnostic certainty.

#### 2.9.1 Ordered image-to-image variability

To characterize variation in model output among images associated with the same infant and laterality, predictions were grouped by infant identifier and laterality. Within each infant–laterality group, the available images were arranged according to their recorded sequence index. Adjacent available images in the resulting order were then paired for descriptive comparison.

Pairs were formed only between images belonging to the same infant and the same laterality. Images from different infants or different laterality groups were never combined. The image order was used only to organize the available observations and was not interpreted as verified acquisition timing, elapsed clinical time, or biological disease progression.

For each infant, the standard deviation and range of all image-level predicted plus-disease probabilities were calculated. Image-to-image variation was additionally summarized using the mean absolute change between adjacent available images, as defined in Equation 10:

$$\Delta_{\mathrm{adj}}=\frac{1}{M}\sum_{g=1}^{G}\sum_{\;j=1}^{n_{g}-1}\left|p_{g,j+1}-p_{g,j}\right|,$$ (10)

where g identifies an infant–laterality group containing at least two images, G is the number of such groups, $p_{g,j}$ is the predicted plus-disease probability for the jth available image in group g, $n_{g}$ is the number of images in that group, and

$$M=\sum_{g=1}^{G}(n_{g}-1)$$ (11)

is the total number of adjacent image pairs, (M), is defined in Equation 11.

In the primary hold-out set, 13 infant–laterality groups contained at least two images and contributed a total of 84 adjacent image pairs. One additional infant–laterality group contained only one image and therefore contributed no pair.

The maximum absolute change between adjacent available images was also recorded. A threshold crossing was defined as a pair in which one predicted probability was below 0.5 and the other was greater than or equal to 0.5. The number of correctly classified individual images was reported for each infant to illustrate how the selection of one available image could affect the resulting classification.

These analyses described variation in model output among ordered images from the same infant and laterality group. They were not interpreted as evidence of temporal or biological changes in disease severity. Differences between images could reflect variation in posterior-pole coverage, focus, illumination, camera angle, retinal visibility, or other image-acquisition characteristics.

To assess whether the observed within-infant variability was specific to DenseNet121, the same descriptive variability analysis was repeated across all five primary CNN architectures. For each architecture, hold-out predictions were grouped by infant and eye laterality, and images within each infant–laterality group were ordered by the same recorded sequence index.

The same 84 adjacent image pairs were therefore evaluated for each architecture. For each model, the mean, median, and maximum absolute probability change between adjacent images were calculated, together with the number and proportion of adjacent pairs crossing the fixed decision threshold of 0.5. Within-infant probability standard deviations were also summarized. These cross-architecture analyses were descriptive and were not used to establish statistical superiority among the models.

### 2.10 Reporting guidelines

This study was reported with reference to the CLAIM 2024 (16) and STARD-AI (26) reporting frameworks. Completed checklists with page and line references to the revised manuscript are provided as Supplementary Files. Items for which the corresponding information was not applicable or was unavailable in the retrospective dataset are identified explicitly in the respective checklists rather than being retrospectively inferred.

## 3 Results

### 3.1 Primary patient-disjoint image-level performance

The patient-disjoint hold-out set comprised 98 images from eight infants, including 51 plus-labelled and 47 non-plus-labelled images. All images from the same infant were restricted to one data partition; however, predictions and performance measures were calculated for individual images.

Performance at the fixed threshold of 0.5 is summarized in Table 2. No architecture was optimal across all reported outcomes. DenseNet121 achieved the highest ROC-AUC of 0.929. MobileNetV3 Large achieved the highest PR-AUC of 0.944, specificity of 1.000, positive predictive value of 1.000, and the lowest Brier score of 0.140. ResNet-18 achieved the highest sensitivity of 0.667.

**Table 2:** Primary patient-disjoint image-level performance on the 98-image hold-out set at the fixed threshold of 0.5.

Model	Acc.	Sens.	Spec.	PPV	NPV	F1	Bal. acc.	ROC-AUC	PR-AUC	Brier
DenseNet121	0.806	0.647	0.979	0.971	0.719	0.776	0.813	**0.929**	0.942	0.148
EfficientNet-B0	0.735	0.549	0.936	0.903	0.657	0.683	0.743	0.861	0.884	0.191
MobileNetV3 Large	0.806	0.627	**1.000**	**1.000**	0.712	0.771	**0.814**	0.927	**0.944**	**0.140**
ResNet-18	0.786	**0.667**	0.915	0.895	0.717	0.764	0.791	0.869	0.889	0.164
ResNet50	0.806	0.647	0.979	0.971	0.719	0.776	0.813	0.904	0.921	0.156

Acc., accuracy; Sens., sensitivity; Spec., specificity; PPV, positive predictive value; NPV, negative predictive value; Bal. acc., balanced accuracy. Plus disease was treated as the positive class. Bold values identify the numerical maximum, or minimum for the Brier score, and do not establish statistical superiority. All performance measures were calculated at the image level.

The five architectures were treated as parallel primary models. Numerical differences in hold-out performance were interpreted descriptively and were not used to establish statistical superiority or to select a definitive architecture.

At the fixed threshold of 0.5, DenseNet121 generated 33 true-positive, 46 true-negative, one false-positive, and 18 false-negative image-level predictions. Its accuracy was 80.6%, sensitivity 64.7%, specificity 97.9%, positive predictive value 97.1%, negative predictive value 71.9%, F1-score 0.776, and balanced accuracy 0.813.

The high specificity and positive predictive value indicate a low false-positive burden within the available hold-out sample. However, 18 of the 51 plus-labelled images were classified as non-plus. Because the 98 image-level observations originated from only eight infants, these values should not be interpreted as results from 98 independent clinical cases or as patient-level diagnostic performance. Confidence intervals assuming independent images were not reported, and the findings are presented descriptively.

The threshold of 0.5 was retained as the reproducible reference operating point. Because this threshold was not selected for a screening-oriented clinical objective, an additional operating point was defined exclusively from development data and evaluated on the patient-disjoint hold-out set, as reported below.

An exploratory training-fraction analysis was also performed using ResNet-18 within the primary patient-disjoint framework. Performance was not monotonic across all metrics, and the experiment was based on a single subsampling realization. The complete results are reported in Supplementary Table S3.

#### 3.1.1 Infant-cluster sensitivity analysis

Because the 98 hold-out images originated from only eight infants and the number of images per infant was unequal, additional cluster-aware sensitivity analyses were performed.

When each infant was assigned equal total weight, ROC-AUC changed from 0.861 to 0.800 for EfficientNet-B0, from 0.929 to 0.905 for DenseNet121, from 0.904 to 0.899 for ResNet50, from 0.927 to 0.920 for MobileNetV3 Large, and from 0.869 to 0.787 for ResNet18. The effect was more pronounced for sensitivity. Conventional vs. infant-equal sensitivity was 0.549 vs. 0.356 for EfficientNet-B0, 0.647 vs. 0.394 for DenseNet121, 0.647 vs. 0.406 for ResNet50, 0.627 vs. 0.389 for MobileNetV3 Large, and 0.667 vs. 0.411 for ResNet18.

The leave-one-infant-out analysis demonstrated additional dependence on cohort composition. ROC-AUC ranged from 0.804 to 0.914 for EfficientNet-B0, 0.838 to 0.989 for DenseNet121, 0.797 to 0.978 for ResNet50, 0.830 to 0.994 for MobileNetV3 Large, and 0.721 to 0.962 for ResNet18 (Supplementary Figure S4).

In the 10,000-resample stratified infant-cluster bootstrap, the exploratory 95% percentile intervals for image-level ROC-AUC were 0.668–0.944 for EfficientNet-B0, 0.752–0.999 for DenseNet121, 0.701–0.997 for ResNet50, 0.769–0.999 for MobileNetV3 Large, and 0.588–0.991 for ResNet18. The width of these intervals reflects the limited number of independent infant clusters. Accordingly, the full-cohort ROC-AUC values should be regarded as descriptive point estimates rather than precise estimates of clinical diagnostic performance. Detailed results of the infant-equal weighting, leave-one-infant-out analysis, and stratified infant-cluster bootstrap are provided in Supplementary Table S5.

#### 3.1.2 Development-defined high-sensitivity operating point

The development-defined sensitivity-oriented analysis demonstrated a marked sensitivity–specificity trade-off across all five architectures. For DenseNet121, the threshold selected exclusively from development out-of-fold predictions was 0.0184. At the reference threshold of 0.5, DenseNet121 achieved a hold-out sensitivity of 64.7% and specificity of 97.9%, with 18 false negatives and one false positive. At the development-defined threshold of 0.0184, hold-out sensitivity increased to 98.0% and false negatives decreased to one, whereas specificity decreased to 46.8% and false positives increased to 25. The proportion of hold-out images classified as plus increased from 34.7% (34/98) to 76.5% (75/98).

Similar sensitivity–specificity trade-offs were observed across the other architectures. EfficientNet-B0 and MobileNetV3 Large achieved 100% hold-out sensitivity at their development-defined thresholds, with specificities of 0.0% and 34.0%, respectively. ResNet50 achieved 96.1% sensitivity and 42.6% specificity, whereas ResNet18 achieved 94.1% sensitivity and 38.3% specificity. These operating points are presented as development-defined sensitivity-oriented analyses and should not be interpreted as clinically validated thresholds. Complete cross-architecture results are provided in Supplementary Table S7.

### 3.2 Descriptive within-infant prediction variability

Grouping the 98 primary hold-out images according to the available infant identifier identified eight infants: three with a plus reference class and five with a non-plus reference class. The number of images available per infant ranged from 1 to 30. Seven infants contributed multiple images, six contributed images from both eyes, one contributed right-eye images only, and one contributed a single left-eye image. Table 3 summarizes the within-infant distribution of DenseNet121 predicted plus-disease probabilities.

**Table 3:** Infant-grouped descriptive summary of DenseNet121 image-level predicted plus-disease probabilities in the primary hold-out set.

Infant	Reference	Images (L/R)	Mean ± SD	Median	Range	Images ≥0.5	Mean probability L/R
P01	Plus	20 (10/10)	0.312±0.256	0.265	0.015–0.892	5/20	0.228/0.396
P02	Plus	30 (15/15)	0.802±0.199	0.868	0.167–0.999	28/30	0.758/0.846
P03	Plus	1 (1/0)	0.294	0.294	0.294–0.294	0/1	0.294/–
P04	Non-plus	8 (4/4)	0.030±0.058	0.011	0.000–0.171	0/8	0.006/0.054
P05	Non-plus	10 (5/5)	0.037±0.045	0.007	0.001–0.124	0/10	0.047/0.027
P06	Non-plus	10 (4/6)	0.029±0.037	0.012	0.000–0.115	0/10	0.006/0.044
P07	Non-plus	9 (4/5)	0.249±0.163	0.302	0.020–0.523	1/9	0.340/0.175
P08	Non-plus	10 (0/10)	0.060±0.086	0.017	0.001–0.212	0/10	–/0.060

L, left eye; R, right eye; SD, standard deviation. Each probability was calculated for one fundus image. The SD was not estimable for P03 because only one image was available. Eye-specific values are descriptive and do not establish inter-eye differences in disease severity.

Substantial variation was observed among images from the same plus-reference infant. For P01, predicted probabilities ranged from 0.015 to 0.892, and only 5 of 20 images exceeded the fixed threshold of 0.5. Its right-eye mean probability was numerically higher than its left-eye mean probability, at 0.396 vs. 0.228. However, considerable variation was present within both eyes.

P02 showed a more consistent high-probability pattern. Twenty-eight of its 30 images exceeded the threshold of 0.5, with a mean probability of 0.802 and a median of 0.868. Mean probabilities were 0.758 for the left eye and 0.846 for the right eye. P03 contributed only one left-eye image, which received a predicted plus-disease probability of 0.294. Within-infant variability could not be estimated for P03 because only one image was available.

Four of the five non-plus-reference infants had no image-level probability above 0.5. P04, P05, and P06 showed consistently low probabilities across the available images. P08 also remained below the threshold across all 10 right-eye images. P07 showed greater within-infant variability, with probabilities ranging from 0.020 to 0.523. One of its nine images exceeded the threshold, and its left-eye mean was numerically higher than its right-eye mean, at 0.340 vs. 0.175.

Figure 1 illustrates the distribution of image-level probabilities within each infant and between the available eyes.

**Figure 1:**
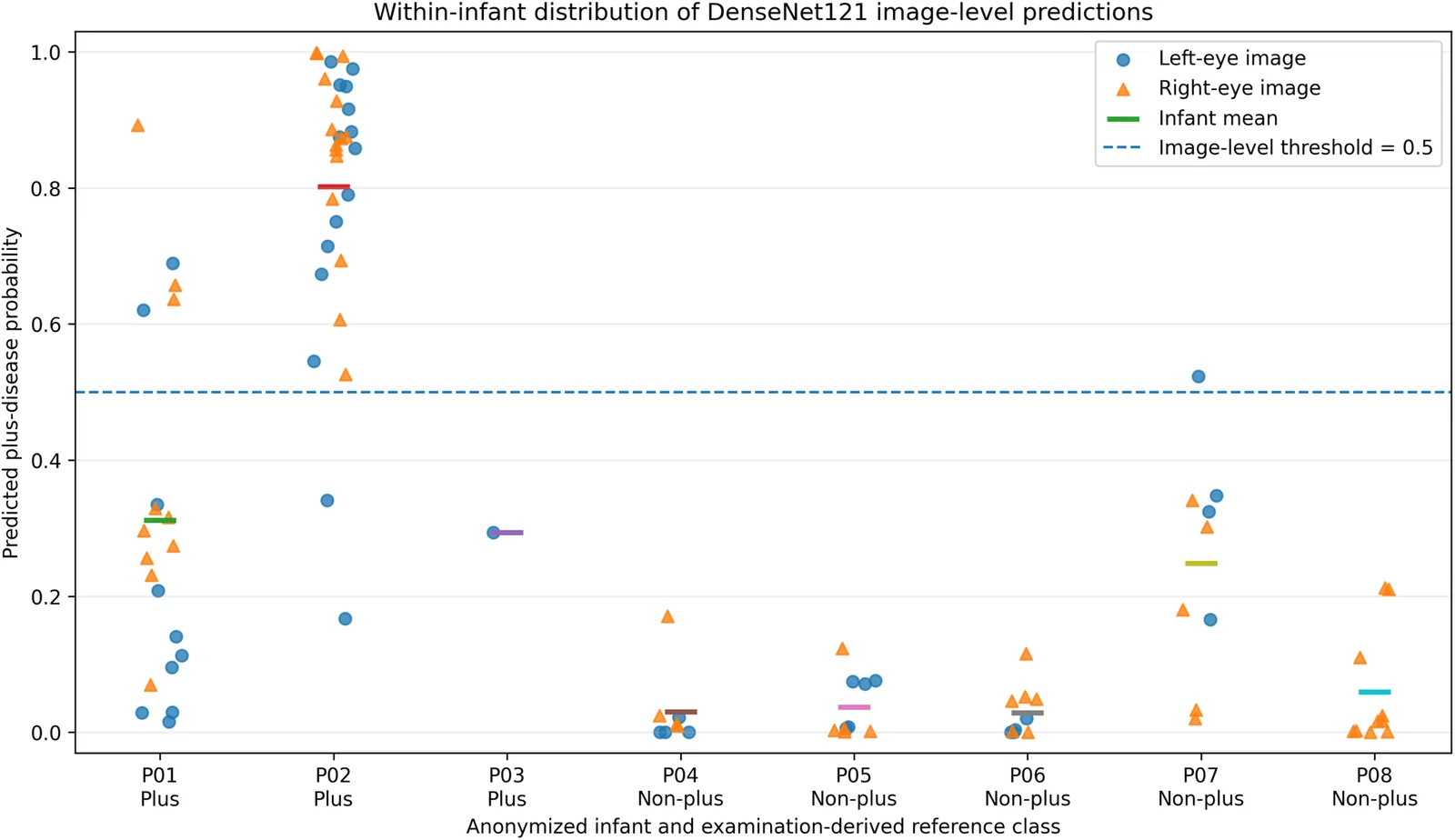
Within-infant distributions of DenseNet121 image-level predicted plus-disease probabilities in the primary patient-disjoint hold-out set. Each point represents one fundus image. Circles represent left-eye images, triangles represent right-eye images, and horizontal markers indicate the mean probability for each infant. The dashed horizontal line represents the fixed image-level decision threshold of 0.5. Infants are ordered as P01–P08 according to the prespecified anonymized mapping. The figure demonstrates variation among images from the same infant and should not be interpreted as validated patient-level diagnostic performance.

Descriptive results of the post hoc aggregation rules are provided in Supplementary Table S2. Because complete examination identifiers were unavailable, these aggregation results were not interpreted as infant- or examination-level diagnostic performance.

#### 3.2.1 Ordered image-to-image variation

Image-level probabilities varied within several infant–laterality groups and, in some cases, crossed the fixed classification threshold of 0.5. Across 84 ordered adjacent image pairs contributed by 13 infant–laterality groups containing at least two images, the mean absolute change in predicted probability was 0.142. One additional infant–laterality group contained only one image and contributed no pair. Eleven pairs crossed the decision threshold. These findings demonstrate that two images adjacent in the filename-ordered sequence for a given infant and laterality could receive substantially different probabilities and, in some cases, different binary classifications.

Table 4 summarizes the ordered image-to-image variability. P01 showed marked variation: predicted probabilities ranged from 0.015 to 0.892, the mean absolute change across its ordered adjacent image pairs was 0.248, and seven such pairs crossed the threshold. Only 5 of 20 images from this plus-reference infant were individually classified as plus disease. Thus, selection of one available image from P01 would have produced the reference-consistent classification in only 25.0% of the available frames.

**Table 4:** Ordered image-to-image variability in DenseNet121 predicted plus-disease probabilities within the primary hold-out set.

Infant	Reference	Images	Probability SD	Probability range	Mean adjacent |Δ|	Threshold crossings	Correctly classified images
P01	Plus	20	0.256	0.015–0.892	0.248	7	5/20 (25.0%)
P02	Plus	30	0.199	0.167–0.999	0.152	2	28/30 (93.3%)
P03	Plus	1	–	0.294–0.294	–	–	0/1 (0.0%)
P04	Non-plus	8	0.058	0.000–0.171	0.060	0	8/8 (100.0%)
P05	Non-plus	10	0.045	0.001–0.124	0.049	0	10/10 (100.0%)
P06	Non-plus	10	0.037	0.000–0.115	0.026	0	10/10 (100.0%)
P07	Non-plus	9	0.163	0.020–0.523	0.192	2	8/9 (88.9%)
P08	Non-plus	10	0.086	0.001–0.212	0.098	0	10/10 (100.0%)

SD, standard deviation; |Δ|, absolute difference in predicted plus-disease probability between ordered adjacent images after arranging the available images by the recorded image sequence index within each infant–laterality group. Threshold crossings indicate ordered adjacent image pairs for which the binary prediction changed across the fixed threshold of 0.5. The 84 pairs were formed within 13 infant–laterality groups containing at least two images and were then pooled. One additional non-empty infant–laterality group contained a single image and contributed no pair. Adjacency in the recorded image order was not interpreted as verified temporal adjacency or as evidence of biological changes in disease severity.

P02 showed a predominantly high-probability pattern, with 28 of 30 images classified as plus. Nevertheless, its probabilities ranged from 0.167 to 0.999, and the largest change between adjacent available images in its ordered laterality groups was 0.707. P03 had only one available image and therefore did not permit evaluation of ordered image-to-image variability.

The non-plus-reference infants P04, P05, P06, and P08 were classified consistently across all available images. P07 showed greater variation: one of nine images was classified as plus, and two ordered adjacent image pairs crossed the threshold. Consequently, selection of that single atypical image would have produced a false-positive result, whereas selection of any of the other eight images would have produced the reference-consistent non-plus classification.

Figure 2 visualizes predicted probabilities according to the recorded image sequence index within each available infant–laterality group.

**Figure 2:**
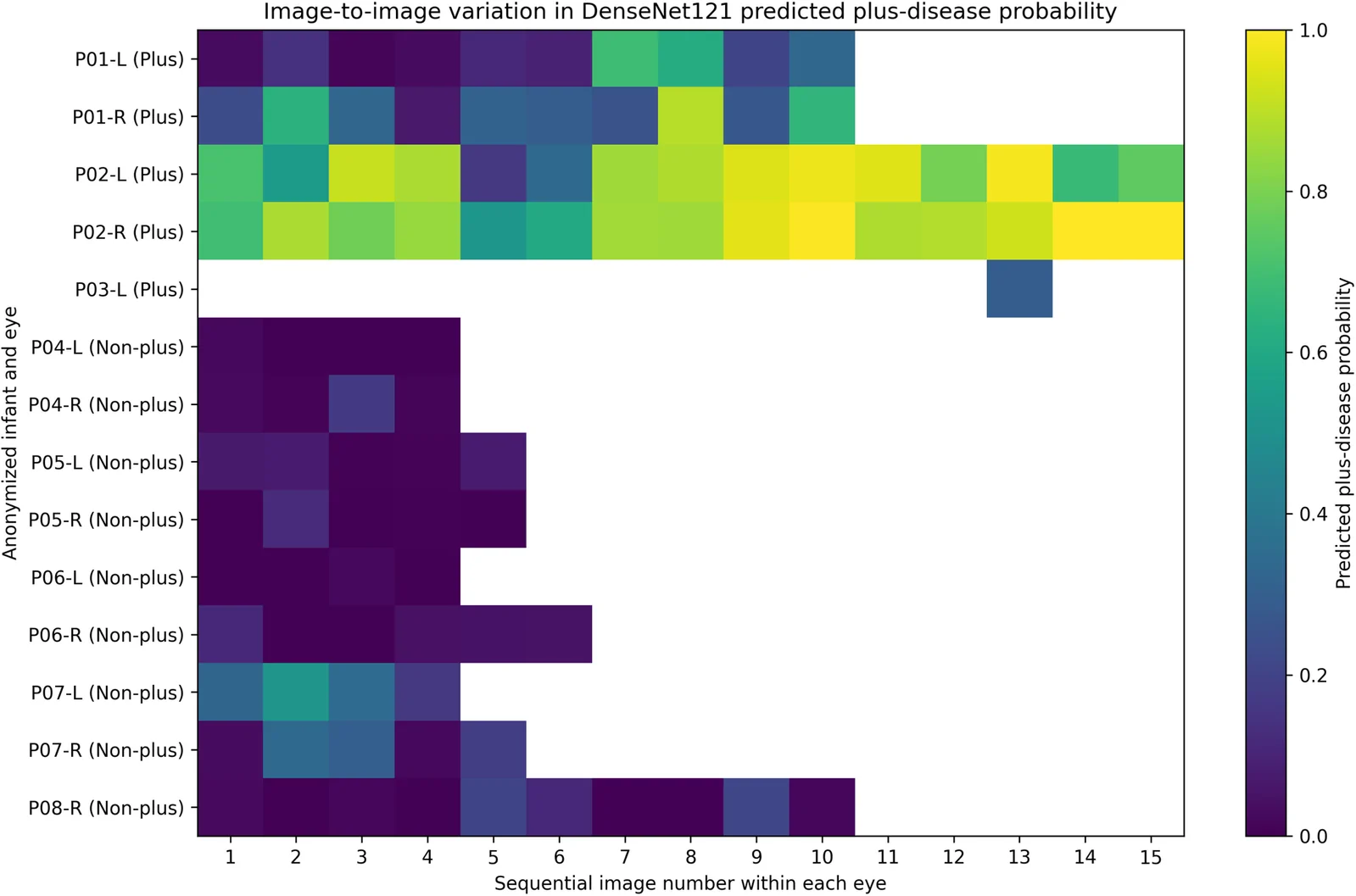
Ordered image-to-image variation in DenseNet121 predicted plus-disease probabilities. Each row represents a separate infant–laterality group, and the columns represent the recorded image sequence index. The colour scale represents the predicted probability of plus disease from 0 to 1. White cells indicate that no image with the corresponding recorded sequence index was available. Marked variation within selected infant–laterality groups for P01, P02, and P07 demonstrates that images associated with the same infant and laterality may generate substantially different model outputs. The recorded image order was used only to organize the available images and was not interpreted as verified temporal acquisition order or biological disease progression.

#### 3.2.2 Cross-architecture consistency of within-infant variability

Repeating the same within-infant analysis across all five architectures demonstrated that image-to-image variability was not specific to DenseNet121. Across the same 84 adjacent image pairs, mean absolute changes in predicted plus-disease probability were 0.171 for EfficientNet-B0, 0.142 for DenseNet121, 0.148 for ResNet50, 0.163 for MobileNetV3 Large, and 0.137 for ResNet18. The corresponding maximum adjacent changes were 0.783, 0.708, 0.883, 0.736, and 0.668, respectively.

Threshold crossings at the fixed decision threshold of 0.5 occurred in 18/84 pairs (21.4%) for EfficientNet-B0, 11/84 (13.1%) for DenseNet121, 18/84 (21.4%) for ResNet50, 12/84 (14.3%) for MobileNetV3 Large, and 11/84 (13.1%) for ResNet18. The mean within-infant probability SD was 0.164, 0.121, 0.142, 0.139, and 0.168, respectively.

Thus, all five architectures exhibited within-infant probability variation and threshold-crossing behaviour. DenseNet121 did not show the largest variability, indicating that the observed dependence on image selection was not unique to the architecture originally highlighted for detailed illustration. Detailed cross-architecture results are provided in Supplementary Table S6.

### 3.3 Exploratory secondary image-level architecture comparison

The historical secondary image-level test set contained 83 images: 47 plus-labelled and 36 non-plus-labelled images. Because patient identity was not enforced during partitioning and complete historical infant-level provenance could not be reconstructed, all results in this section are interpreted as exploratory image-level findings within the available historical collection. They are not considered independent patient-level validation and should not be compared directly with the primary patient-disjoint analysis as equivalent evidence of generalization. Performance of the five architectures on the secondary 83-image test set is summarized in Table 5. ResNet-18 and DenseNet121 produced nearly identical test confusion patterns, while EfficientNet-B0 achieved the highest numerical PPV and tied with MobileNetV3 Large for the highest specificity among the lighter models. Because images were the unit of splitting, these figures describe held-out image performance and not independent patient-level performance.

**Table 5:** Exploratory performance of five CNN architectures in the historical secondary random image-level analysis.

Model	Parameters (M)	Accuracy	PPV	Sensitivity	Specificity	F1
EfficientNet-B0	5.3	0.939	**0.956**	0.935	**0.944**	0.945
DenseNet121	8.0	0.939	0.936	**0.957**	0.917	0.946
ResNet50	25.6	0.915	0.915	0.935	0.889	0.925
MobileNetV3 Large	5.4	0.927	0.955	0.913	**0.944**	0.933
ResNet-18	11.7	**0.940**	0.938	**0.957**	0.917	**0.947**

Patient separation was not enforced; therefore, these results represent supportive image-level findings and not independent patient-level validation. Bold values indicate the numerical maximum within each metric column.

For ResNet-18, the confusion matrix comprised 45 true positives, 33 true negatives, 3 false positives, and 2 false negatives. Accuracy was 94.0% (95% CI, 86.7–97.4%), sensitivity 95.7% (85.8–98.8%), specificity 91.7% (78.2–97.1%), PPV 93.8% (83.2–97.9%), NPV 94.3% (81.4–98.4%), F1-score 0.947, and MCC 0.877. ROC-AUC was 0.974 and the Brier score was 0.0573. These numerical estimates should not be interpreted as evidence of independent patient-level performance because the historical partition was constructed at the image level and may contain correlated images from the same infant across data partitions.

An exploratory training-fraction analysis was also performed for ResNet-18 within the secondary random image-level framework. Performance generally increased as more training images were used, although the results were based on one subsampling realization and were not monotonic across all measures. Complete results are reported in Supplementary Table S4.

### 3.4 Exploratory image-level explainability and error analysis

Selected image-level Grad-CAM examples from the exploratory secondary analysis are shown in Figure 3. In the two true-positive examples, activation overlapped portions of the major vascular arcades. In the two false-negative examples, activation appeared more diffuse or was concentrated away from the most visually prominent vessels. This pattern is anatomically plausible, but the examples were selected qualitatively and no ophthalmologist-defined localization ground truth was available. The figure therefore supports visual inspection of model behaviour rather than a quantitative claim that the model relied on the same features as an ophthalmologist.

**Figure 3:**
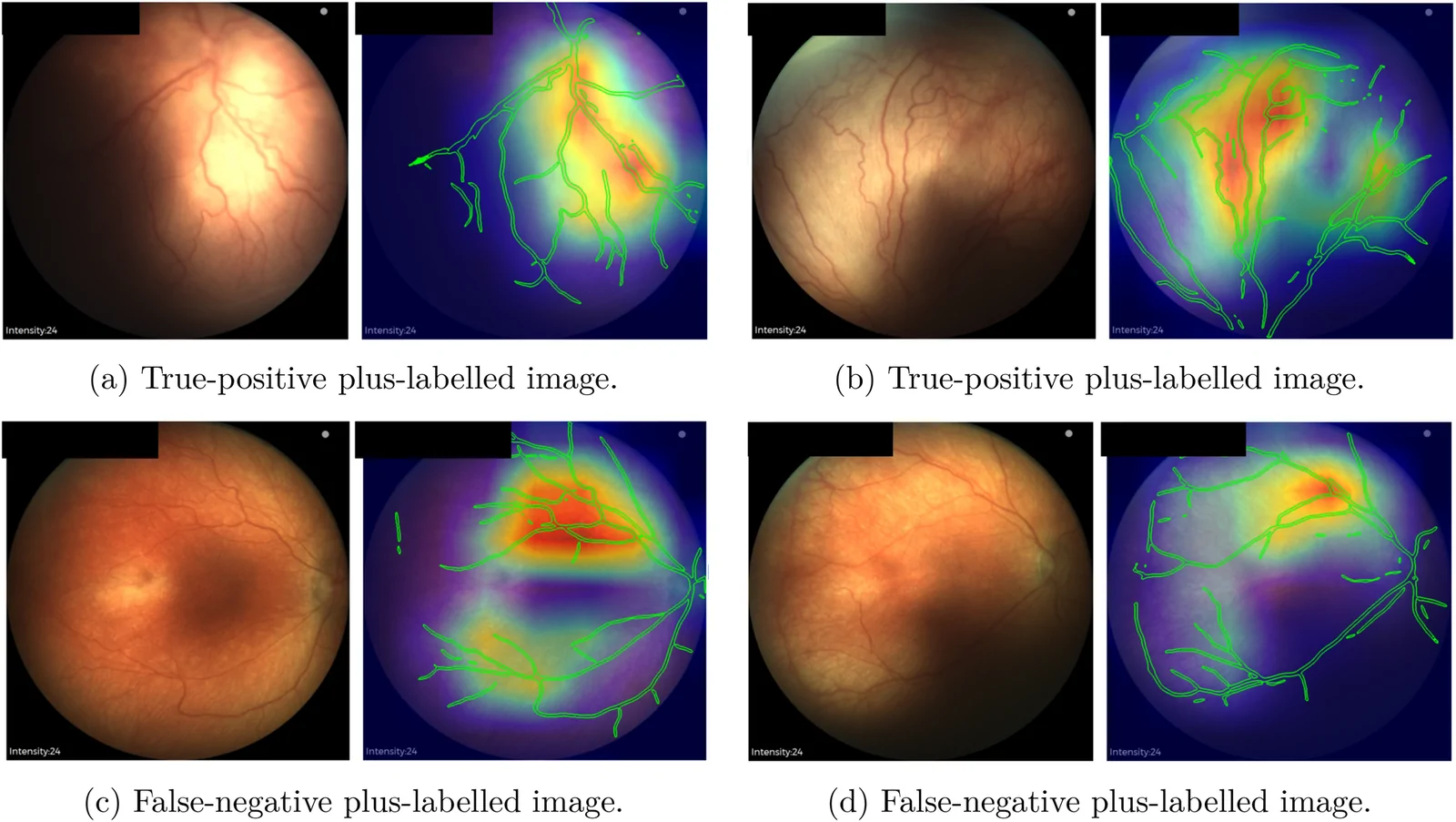
Qualitative Grad-CAM examples from the exploratory secondary image-level analysis. Each panel shows the original fundus image and the corresponding Grad-CAM heatmap overlaid on the RGB fundus image. The heatmaps represent activation maps averaged across the five fold-specific ResNet-18 models and restricted to the retinal region-of-interest. The visualizations are qualitative anatomical plausibility aids and do not establish causal model reasoning, quantitative anatomical localization, or ophthalmologist-equivalent interpretation. **(a)** True-positive plus-labelled image. **(b)** True-positive plus-labelled image. **(c)** False-negative plus-labelled image. **(d)** False-negative plus-labelled image.

As a complementary model-agnostic local explanation, Local Interpretable Model-agnostic Explanations (LIME) (27) was additionally applied to three illustrative fundus images using an ensemble of three available p80 fold-specific ResNet-18 models from the primary patient-disjoint training-fraction analysis. Representative LIME examples are shown in Figure 4. The three illustrative images were selected from the independent patient-disjoint hold-out set and were not used for model fitting or checkpoint selection. Before LIME analysis, the non-retinal black background and acquisition annotations were removed, and only the circular retinal field was retained. The retinal field was partitioned into approximately 30 SLIC superpixels (28), with the exact number varying slightly after retinal-field masking. Superpixels were perturbed and a locally weighted linear surrogate model was fitted to estimate their local contributions to the predicted Plus probability. Positive local coefficients indicate regions supporting the Plus prediction, whereas negative coefficients indicate regions opposing it. The probabilities reported below correspond to the masked retinal inputs used specifically for this LIME analysis. The weighted local surrogate $R^{2}$ is additionally reported for each example as an indicator of the fidelity with which the local linear surrogate approximated the CNN prediction in the neighbourhood of the analyzed image. These visualizations are presented as exploratory local explanations and were not validated against ophthalmologist-defined localization masks.

**Figure 4:**
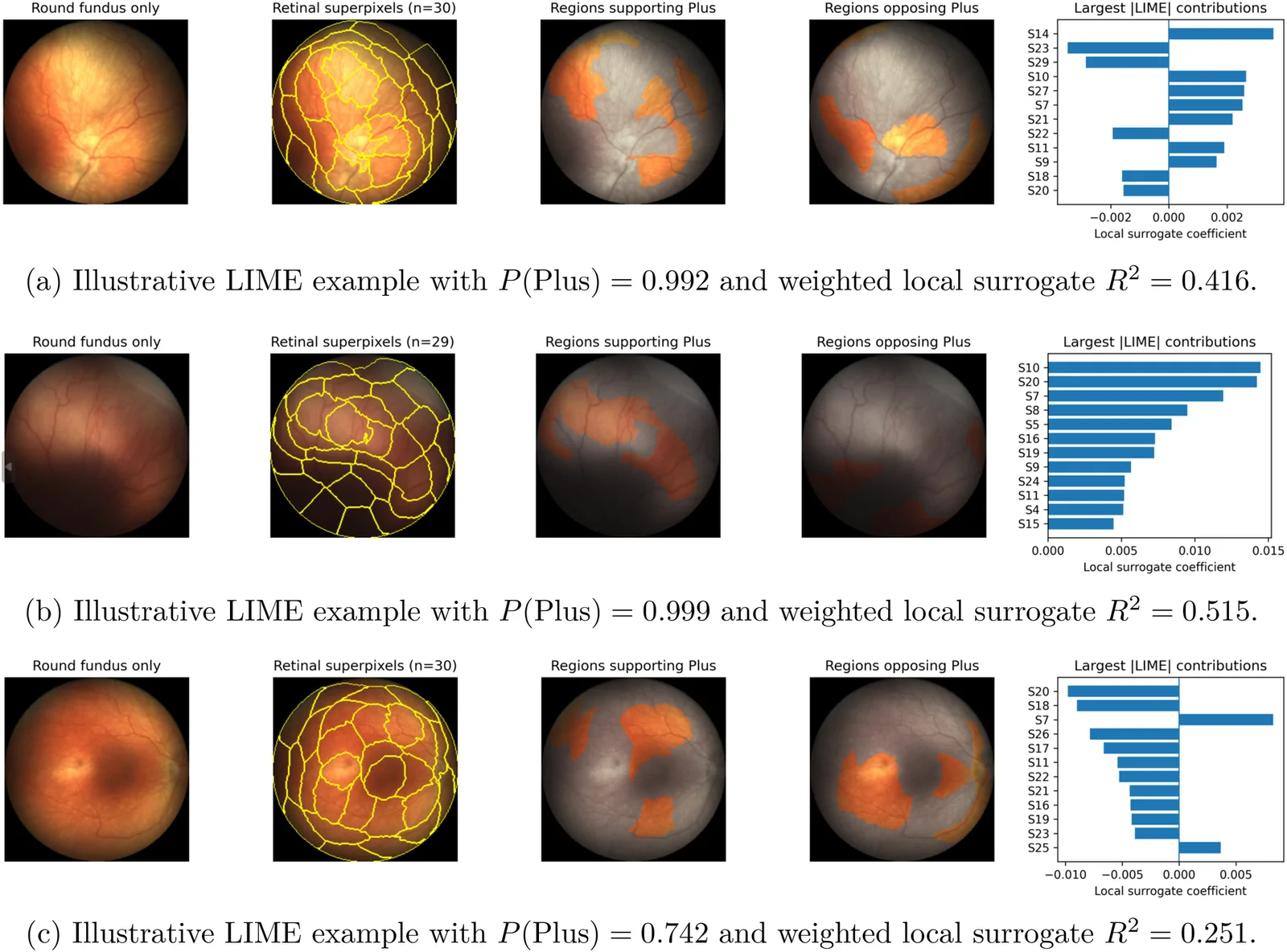
Illustrative model-derived LIME explanations for three fundus images. Each example shows the masked fundus input, retinal superpixel partition, regions locally supporting and opposing the Plus prediction, and the largest local surrogate coefficients. Approximately 30 SLIC superpixels were targeted for each image; the final number varied slightly after retinal-field masking. The weighted local $R^{2}$ values indicate the fidelity of the local linear surrogate to the CNN prediction in the neighbourhood of each analyzed image and demonstrate variable local approximation across examples. The visualizations were not compared with ophthalmologist-defined localization masks and should therefore not be interpreted as quantitative anatomical localization, causal feature attribution, or ophthalmologist-equivalent reasoning. **(a)** Illustrative LIME example with P(Plus)=0.992 and weighted local surrogate $R^{2}=0.416$. **(b)** Illustrative LIME example with P(Plus)=0.999 and weighted local surrogate $R^{2}=0.515$. **(c)** Illustrative LIME example with P(Plus)=0.742 and weighted local surrogate $R^{2}=0.251$.

The image-level probability distribution from the exploratory secondary analysis is shown in Supplementary Figure S1. At threshold 0.5, two of 47 plus-labelled images were false negatives and three of 36 non-plus-labelled images were false positives. Figure 5 shows the two false-negative plus-labelled examples and their class-specific Grad-CAM/vessel overlays. Because no formal image-quality grading protocol was applied, these examples are presented as visually reviewed errors without assigning a proven clinical or technical cause of failure.

**Figure 5:**
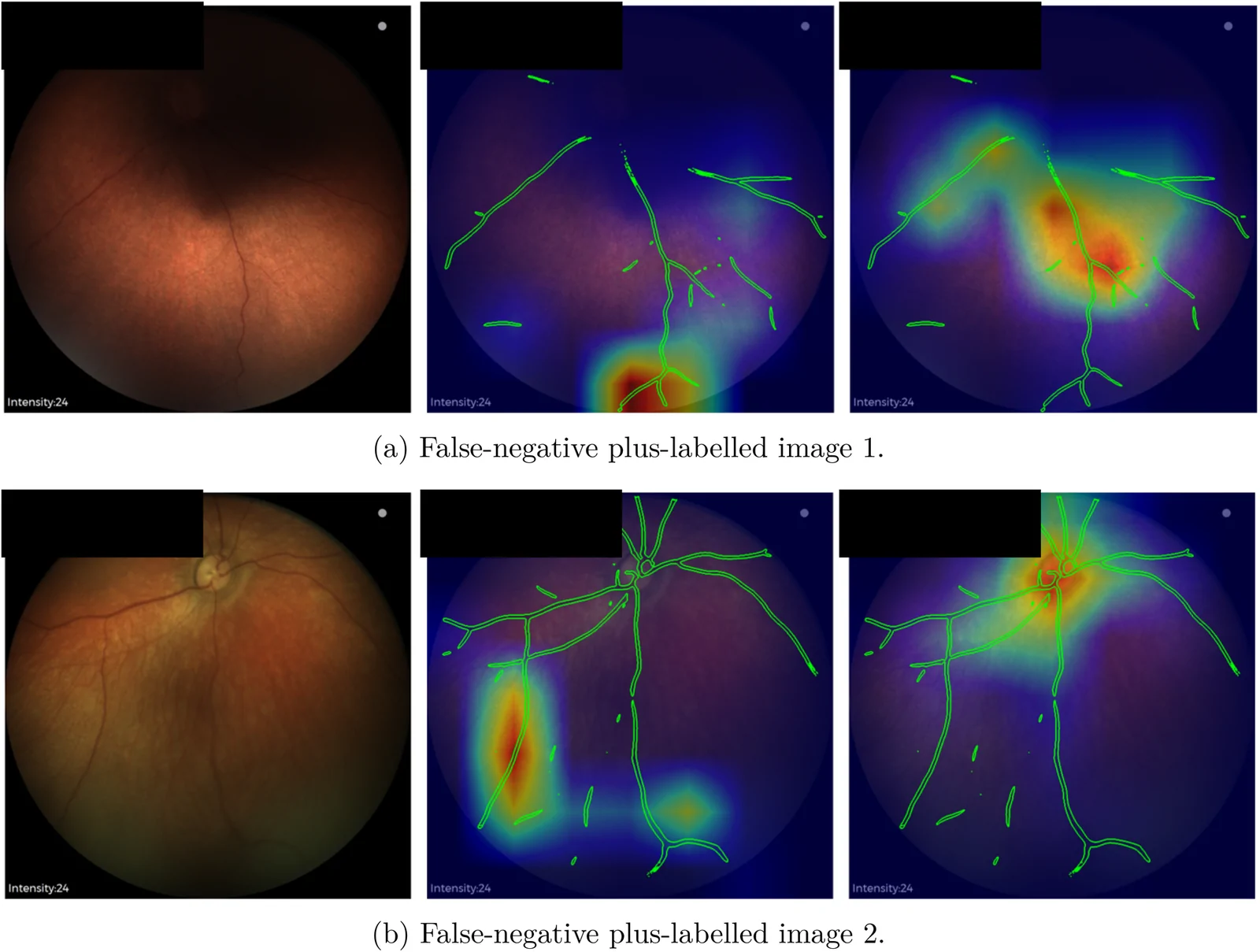
Visual review of the two false-negative image-level cases. Each row shows the original image followed by plus-class and non-plus-class Grad-CAM/vessel overlays. Identifiers were obscured. The images illustrate model error patterns but do not establish the clinical cause of misclassification. **(a)** False-negative plus-labelled image 1. **(b)** False-negative plus-labelled image 2.

The complete Grad-CAM generation, fold averaging, retinal masking, and visualization procedure is described in Supplementary Methods. The LIME examples are presented as an additional exploratory visualization and were not used for model selection or quantitative performance evaluation.

### 3.5 Exploratory secondary image-level probability reliability

The image-level ResNet-18 Brier score was 0.0573. The reliability diagram (Figure 6) showed broad agreement between mean predicted probability and observed plus frequency in the lowest and highest probability regions, while several intermediate bins contained few observations and correspondingly wide Wilson intervals. The result indicates relatively low probabilistic error within this historical image-level test set; however, it does not establish calibration under patient-disjoint or external evaluation.

**Figure 6:**
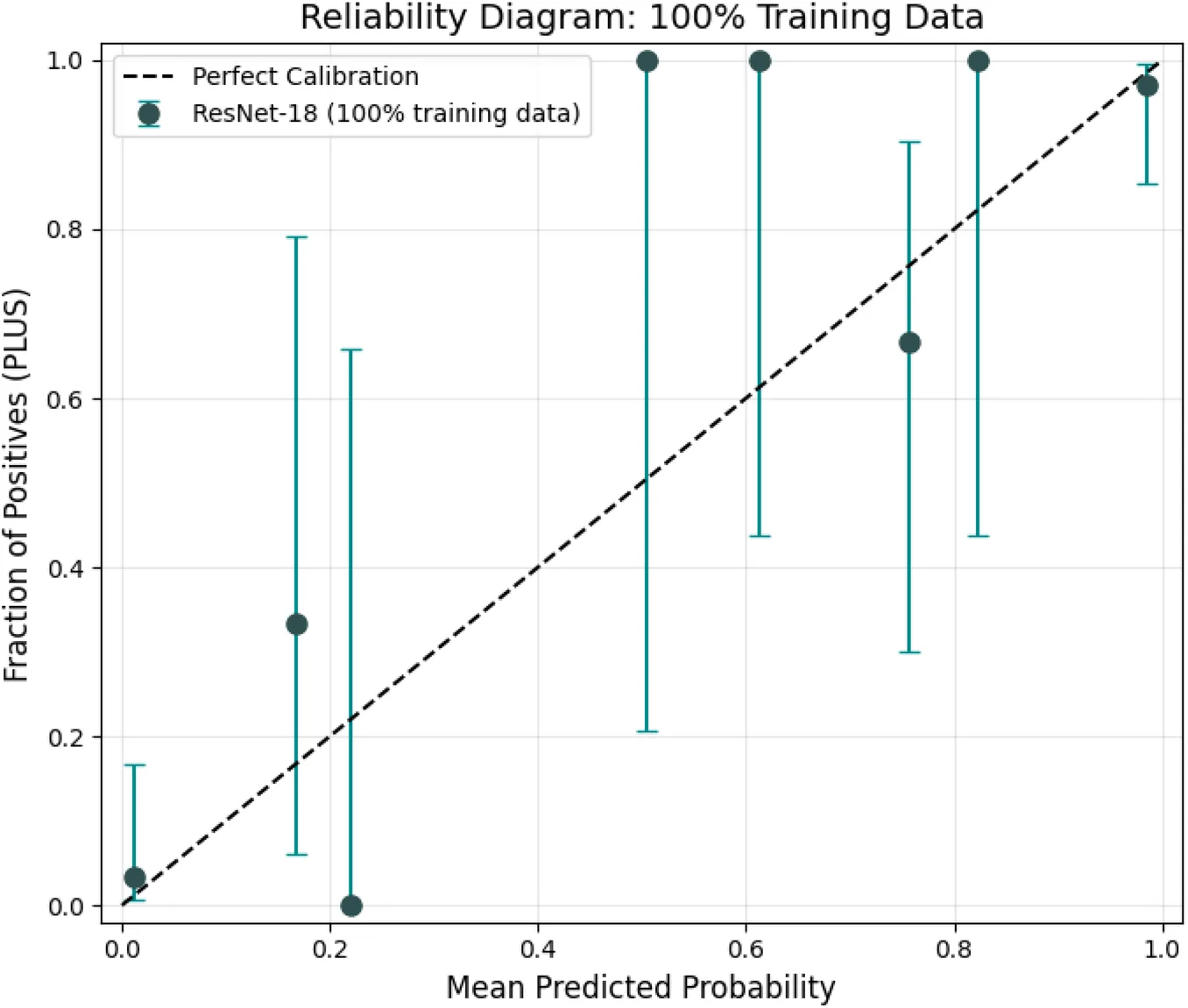
Exploratory reliability diagram for the ResNet-18 fold ensemble evaluated on the historical secondary 83-image test set. Points show observed plus frequency vs. mean predicted probability; vertical bars show Wilson 95% confidence intervals. The dashed line represents perfect calibration. Brier score = 0.0573. Sparse intermediate bins limit precision. Because patient separation was not enforced, this figure does not establish calibration under independent patient-level evaluation.

### 3.6 Exploratory secondary raw-fundus vs. vessel-overlay analysis

To determine whether explicitly emphasizing the retinal vasculature could improve plus-disease classification, an additional image-level experiment compared raw fundus images with vessel-overlay images. The vessel-overlay images were generated from the same raw fundus images using a binary vessel-segmentation mask produced by a separately trained U-Net. The predicted vessels were overlaid in green, while the remaining non-vascular regions of the fundus image were dimmed. The purpose of this representation was to direct the classifier toward vascular abnormalities associated with plus disease, particularly arterial tortuosity and venous dilation, and to assess whether highlighting the segmented vessels improved model performance.

The VGG16 model trained on raw fundus images achieved a ROC-AUC of 0.959, compared with 0.906 for the model trained on vessel-overlay images (Table 6). The raw-fundus model also achieved higher accuracy, sensitivity, and specificity at the reported decision threshold. The corresponding ROC curves and confusion matrices are presented in Supplementary Figures S2 and S3, respectively.

**Table 6:** Image-level VGG16 comparison of raw-fundus and vessel-overlay inputs. This exploratory experiment used the historical secondary image-level framework and is not interpreted as independent patient-level validation.

Input	ROC-AUC	Accuracy	Sensitivity	Specificity	TN	FP	FN	TP
Raw fundus	0.959	0.892	0.851	0.944	34	2	7	40
Vessel overlay	0.906	0.843	0.787	0.917	33	3	10	37

The vessel-overlay representation did not improve classification in this experiment. Because no paired inferential comparison was performed, the difference between the two pipelines is reported descriptively. Moreover, the raw-fundus and vessel-overlay pipelines differed in both input representation and colour-augmentation procedures; therefore, the observed difference cannot be attributed exclusively to vessel highlighting. Potential technical and clinical explanations are considered in Section 4.6.

### 3.7 Single-image ophthalmologist reader study

The reader analysis included 30 unique fundus images: 18 carrying a plus reference label and 12 carrying a non-plus reference label. Ten of the 30 images were presented a second time, resulting in 40 presentations in total. The repeated presentations were excluded from performance and inter-observer analyses and were used only to assess intra-observer repeatability. The reader performance, agreement, and repeatability results are summarized in Table 7.

**Table 7:** Agreement of isolated-image ophthalmologist assessments with examination-derived reference labels and reader repeatability.

Measure	Ophthalmologist 1	Ophthalmologist 2
Strict sensitivity: definite Plus positive	1/18 (5.6%; 95% CI, 0.1–27.3%)	0/18 (0.0%; 95% CI, 0.0–18.5%)
Strict specificity	12/12 (100%; 95% CI, 73.5–100%)	12/12 (100%; 95% CI, 73.5–100%)
Abnormality-sensitive sensitivity: Borderline/Pre-plus or Plus positive	4/18 (22.2%; 95% CI, 6.4–47.6%)	1/18 (5.6%; 95% CI, 0.1–27.3%)
Abnormality-sensitive specificity	12/12 (100%; 95% CI, 73.5–100%)	12/12 (100%; 95% CI, 73.5–100%)
Healthy/Borderline/Pre-plus / Plus assessments	26/3/1	29/1/0
Plus-reference images assessed as Healthy	14/18	17/18
Mean confidence for these Healthy assessments	4.14/5	4.59/5
Mean confidence, all unique images	4.27±1.08	4.70±0.53
Repeated severity-grade agreement	10/10	10/10
Repeated confidence exact agreement	8/10	7/10
Mean absolute repeated-confidence difference	0.2	0.3
Exact three-category inter-observer agreement	26/30 (86.7%)	
Unweighted Cohen’s κ	0.161	
Linear-weighted κ	0.302	
Quadratic-weighted κ	0.478	
Binary abnormality agreement	27/30 (90.0%)	
Binary abnormality Cohen’s κ	0.366	

*Note:* Reference labels were derived from the complete multi-image clinical examination and were inherited by the individual images. Under the strict reader definition, only a definite Plus assessment was considered positive. Under the abnormality-sensitive definition, Borderline/Pre-plus and Plus assessments were considered positive. Exact 95% confidence intervals for sensitivity and specificity were calculated using the Clopper–Pearson method. Repeated presentations were used only to assess intra-observer repeatability. The reader results measure agreement between isolated-image assessments and examination-derived reference labels and should not be interpreted as examination-level diagnostic accuracy.

Ophthalmologist 1 graded 26 images as Healthy, 3 as Borderline/Pre-plus, and 1 as Plus. Ophthalmologist 2 graded 29 images as Healthy, 1 as Borderline/Pre-plus, and none as definite Plus.

When only a definite Plus assessment was considered positive, sensitivity relative to the examination-derived reference labels was 5.6% for Ophthalmologist 1 and 0.0% for Ophthalmologist 2. Specificity was 100% for both ophthalmologists. When both Borderline/Pre-plus and Plus assessments were considered positive, sensitivity was 22.2% for Ophthalmologist 1 and 5.6% for Ophthalmologist 2, while specificity remained 100% for both.

These measures quantify agreement between isolated-image assessments and the examination-derived reference labels. They should not be interpreted as measures of routine ophthalmologist examination-level diagnostic accuracy because the readers assessed one isolated image without access to the remaining views from the corresponding clinical examination.

Figure 7 summarizes the examination-derived reference labels and ophthalmologist assessments for the 30 unique isolated images. It also shows the original and repeated assessments for the 10 repeated images.

**Figure 7:**
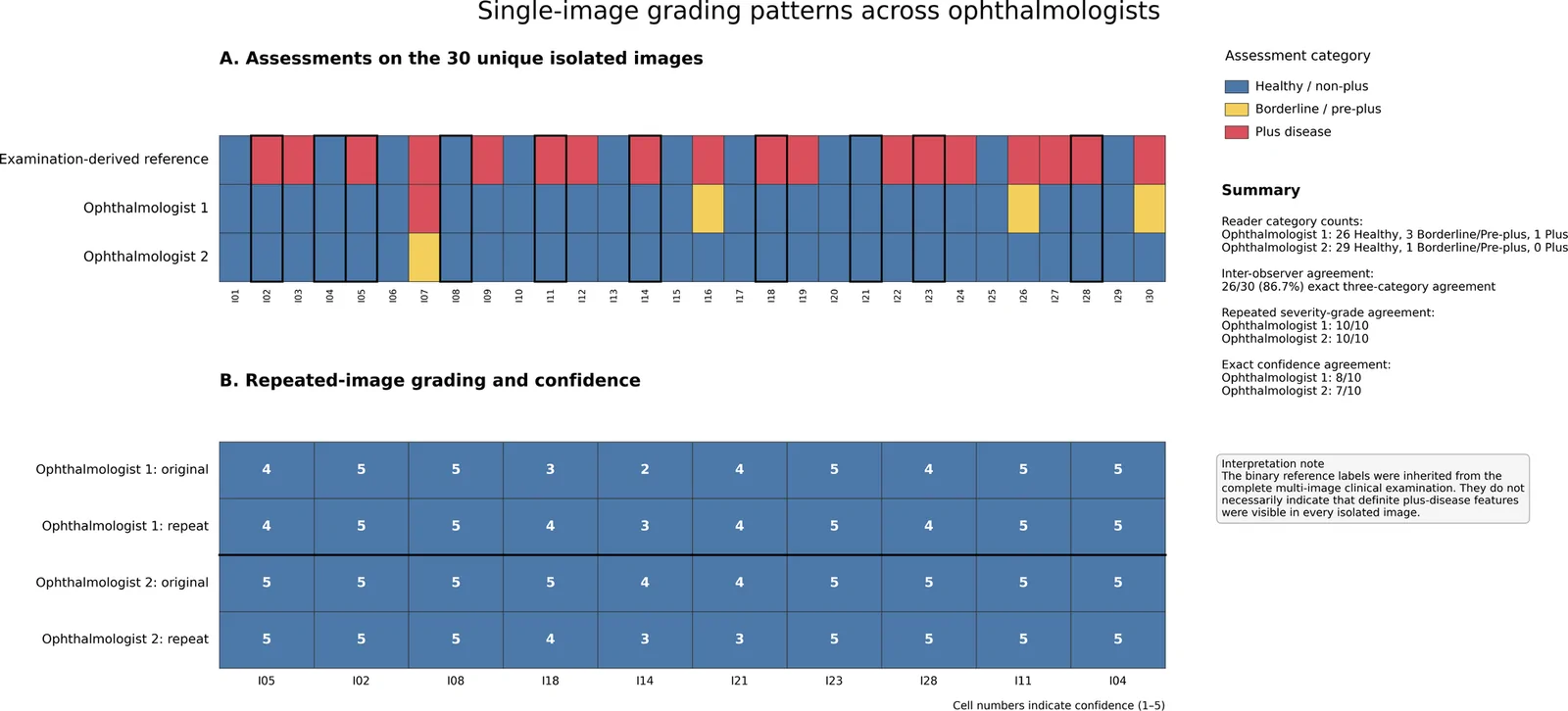
Visual summary of the single-image ophthalmologist reader study. Panel **(A)** shows the examination-derived reference label and the independent assessments of the two ophthalmologists for the 30 unique isolated fundus images. Black-outlined columns identify the 10 images that were presented a second time. Panel **(B)** compares the original and repeated ophthalmologist assessments; numbers within the cells indicate confidence scores on the 1–5 scale. Both ophthalmologists reproduced the same severity grade for all 10 repeated images. Exact confidence agreement was 8/10 for Ophthalmologist 1 and 7/10 for Ophthalmologist 2. The reference labels were derived from the complete multi-image examination and do not necessarily indicate that definite plus-disease features were visible in every isolated image.

Both ophthalmologists reproduced the same severity grade for all 10 repeated images. Exact repeated-confidence agreement was 80% for Ophthalmologist 1 and 70% for Ophthalmologist 2, with mean absolute confidence differences of 0.2 and 0.3 points, respectively. Thus, categorical grading was completely reproducible within the repeated subset, although confidence varied slightly in some presentations.

Both ophthalmologists assigned a Healthy assessment to the same 26 of the 30 unique images. Among these 26 images, the image-level ResNet-18 classifier predicted plus disease for 13 and non-plus for 13. This disagreement does not establish diagnostic superiority of either the model or the ophthalmologists because the model was evaluated against inherited examination-derived labels, whereas the ophthalmologists assessed only the vascular appearance visible in one isolated image.

Exact three-category inter-observer agreement was 86.7%. Unweighted Cohen’s κ was 0.161, linear-weighted κ was 0.302, and quadratic-weighted κ was 0.478. The difference between the high percentage agreement and the lower kappa values reflects the strong prevalence of Healthy assessments by both readers.

After reducing the ophthalmologist assessments to Healthy vs. any vascular abnormality, exact inter-observer agreement was 90.0% and Cohen’s κ was 0.366. Agreement between the binary ResNet-18 prediction and the abnormality-sensitive ophthalmologist assessment was 56.7% for Ophthalmologist 1 (κ=0.211) and 46.7% for Ophthalmologist 2 (κ=0.051).

Most isolated images carrying a plus reference label were assessed as Healthy, frequently with high confidence. This finding supports the distinction between an examination-derived reference diagnosis and the vascular evidence visible within one isolated image. Because image gradability and posterior-pole adequacy were not formally evaluated, the specific reasons for individual disagreements could not be determined.

Full details regarding image selection, presentation randomization, reader blinding, repeat-image placement, reader experience, display conditions, and the provenance of model predictions are provided in the Supplementary Methods.

### 3.8 Illustrative discordance between isolated-image and examination-derived reference

The selected infant had a clinical reference diagnosis of plus disease based on review of the complete multi-image examination. However, the individual fundus images acquired during the same visit differed substantially in their diagnostic content. Some images provided relatively clear visualization of the optic disc and major vascular arcades, whereas others were dark, off-axis, incompletely focused, or showed retinal regions in which the posterior pole was not adequately represented.

Figure 8 provides the visual context for this case. The images were acquired from both eyes during the same clinical examination but varied in illumination, focus, field of view, retinal coverage, and visibility of the posterior pole. Consequently, the diagnostic information available in one isolated image was not necessarily representative of the complete multi-image examination. The image outlined in green was subsequently evaluated by the two ophthalmologists in the single-image reader study.

**Figure 8:**
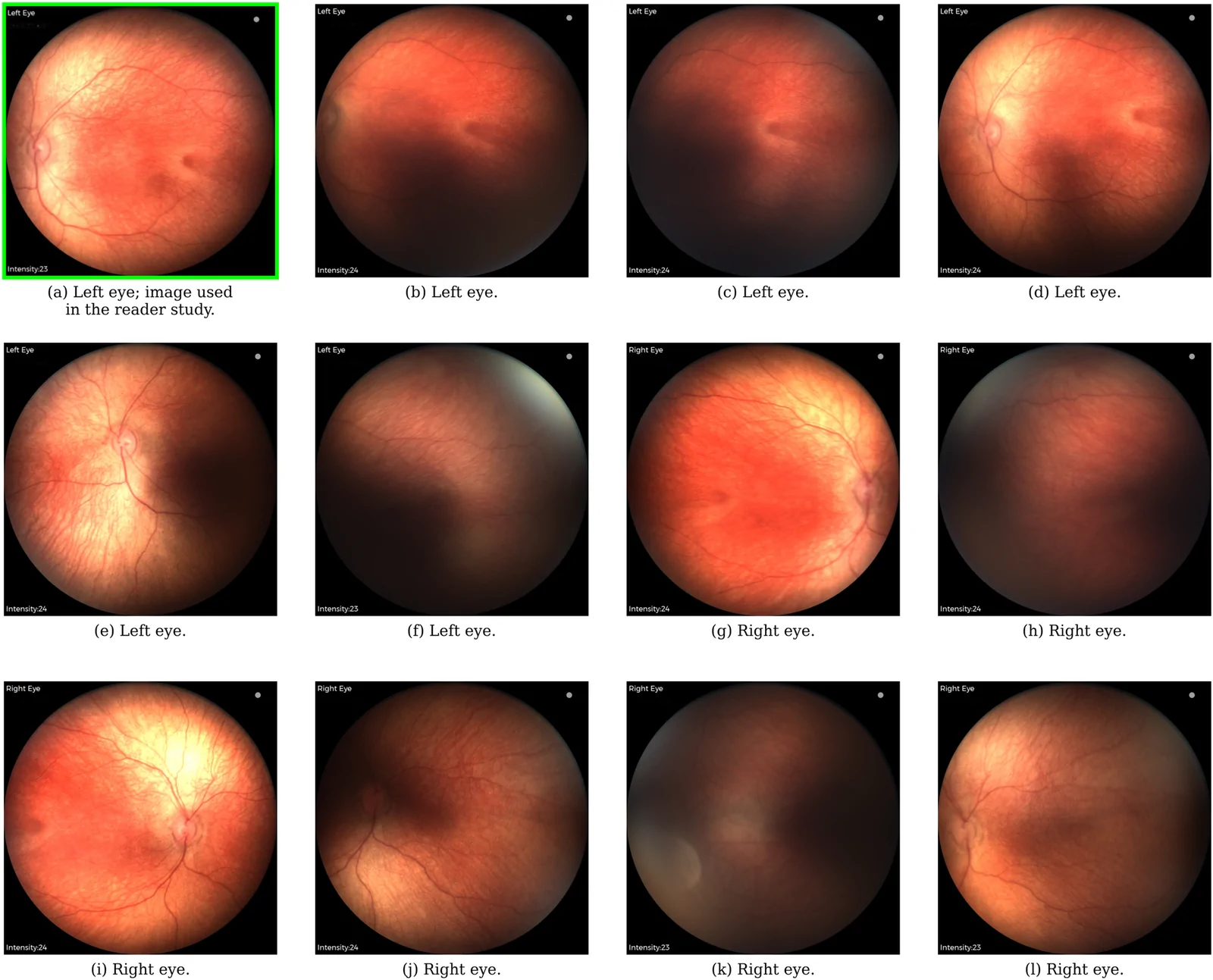
Representative fundus images acquired from both eyes of the same infant during one clinical examination. The images demonstrate substantial within-examination variability in illumination, focus, field of view, retinal coverage, and visibility of the optic disc and posterior-pole vascular arcades. The image outlined in green was included in the ophthalmologist single-image questionnaire. Both ophthalmologists graded this image as Healthy with confidence 5/5, although the clinical reference diagnosis based on the complete multi-image examination was plus disease. The figure illustrates why an isolated image may not contain the same diagnostic information as the complete examination.

The reader-study image highlighted in Figure 8 was independently graded as Healthy by both ophthalmologists, with confidence 5/5 from each reader, whereas the complete examination carried a clinical reference diagnosis of plus disease. This example illustrates that the diagnostic information visible in an isolated frame may differ from that available from the complete multi-image examination.

The high-confidence Healthy assessments should not be interpreted as poor clinical performance by the ophthalmologists. The readers evaluated only one isolated frame without access to the remaining images from the examination, whereas the clinical diagnosis had been established after review of multiple images from both eyes. The findings therefore indicate that the selected frame did not independently provide the same diagnostic information as the complete examination.

The high plus probability produced by the image-level model should also be interpreted cautiously. Agreement between the image-level prediction and the clinical reference diagnosis does not prove that the model identified the posterior-pole vascular dilation and tortuosity used by ophthalmologists. The model may have responded to subtle vascular information, but it may also have relied on non-specific retinal appearance, illumination, camera-related features, or other acquisition characteristics.

The image-level model assigned a relatively high plus-disease probability to the isolated reader-study image, despite both ophthalmologists grading that image as Healthy with maximum confidence. This disagreement should be interpreted cautiously. The model prediction was evaluated against the examination-derived reference label, whereas the ophthalmologists assessed only the vascular information visible in the isolated image. Agreement between the model prediction and the examination-derived label therefore does not establish that definite plus-disease features were independently visible in that frame or that the model relied on clinically valid vascular features.

Figure 9 provides a descriptive visualization of this example. The Grad-CAM map is presented only as a qualitative anatomical plausibility aid and does not demonstrate causal feature use or reasoning equivalent to that of an ophthalmologist.

**Figure 9:**
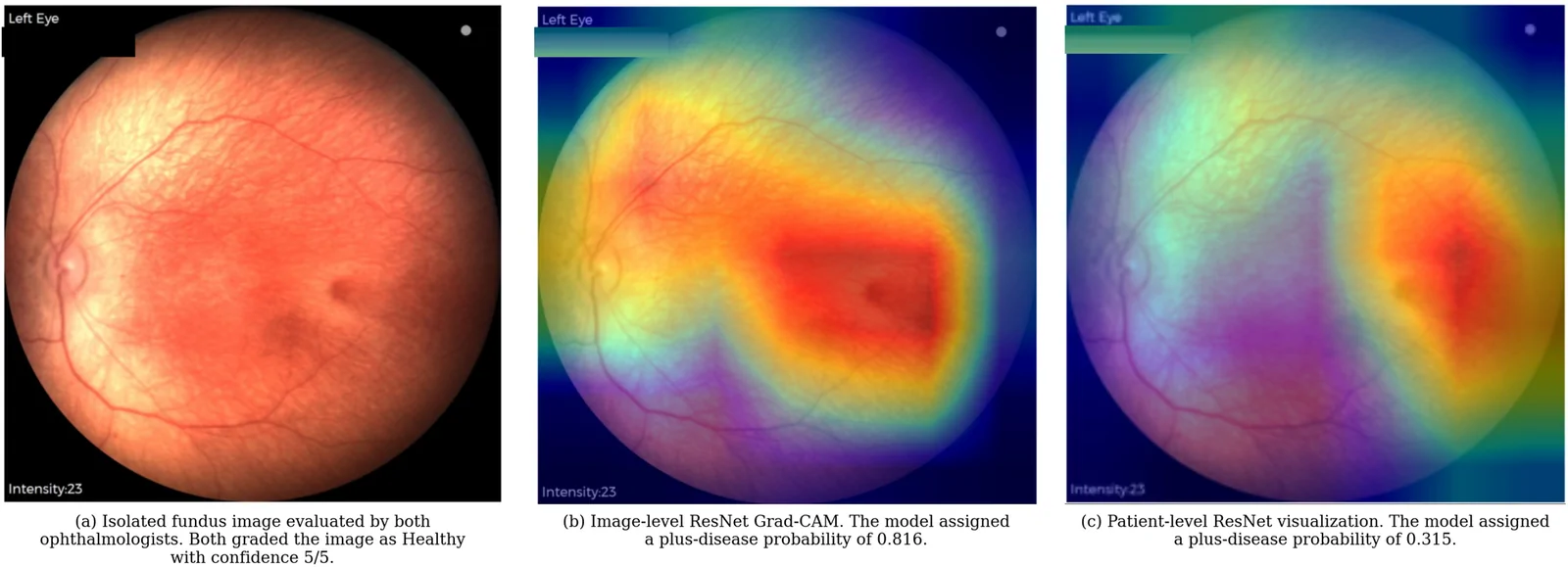
Illustrative comparison between isolated-image ophthalmologist assessment and image-level model output in a clinically diagnosed plus-disease case. The selected isolated fundus image was graded as Healthy with maximum confidence by both ophthalmologists, whereas the image-level model assigned a relatively high plus-disease probability. The clinical reference diagnosis was established from the complete multi-image examination. The Grad-CAM visualization is presented only as a qualitative anatomical plausibility aid and does not establish causal model reasoning or ophthalmologist-equivalent interpretation.

This single illustrative case was not used to estimate diagnostic accuracy or patient- or examination-level model performance. Rather, it demonstrates the distinction between the information visible in one isolated fundus image and the examination-derived clinical reference diagnosis.

## 4 Discussion

### 4.1 Principal findings

This study evaluated deep-learning classification of ROP plus disease using two complementary image-level analytical strategies, together with an ophthalmologist reader study, a vessel-overlay experiment, and qualitative model-visualization analyses. The primary analysis used a patient-disjoint partition, ensuring that all images from each infant were restricted to either the development pool or the hold-out set. This design prevented direct infant overlap between model development and evaluation. However, predictions and performance measures were calculated for individual images: the hold-out set contained 98 images from 8 infants rather than 98 independent patient-level cases. The primary findings should therefore be interpreted as descriptive patient-disjoint image-level performance and not as patient- or examination-level diagnostic accuracy.

No single architecture was optimal across all performance measures. DenseNet121 achieved the highest ROC-AUC of 0.929, MobileNetV3 Large achieved the highest PR-AUC, specificity, and positive predictive value and the lowest Brier score, while ResNet-18 achieved the highest sensitivity at the fixed threshold of 0.5. These differences illustrate that model selection should be guided by the intended clinical role and operating requirements rather than by a single summary metric. The numerical differences among architectures were descriptive and should not be interpreted as evidence of statistical superiority.

DenseNet121 showed a high-specificity operating profile at the fixed threshold of 0.5. It correctly classified 46 of 47 non-plus-labelled images, corresponding to a specificity of 97.9%, and produced only one false-positive prediction. Its positive predictive value was 97.1% within the available hold-out sample. However, the model misclassified 18 of the 51 plus-labelled images as non-plus, resulting in a sensitivity of 64.7%. This false-negative burden is incompatible with autonomous screening, for which failure to identify treatment-relevant disease would be clinically consequential. Moreover, positive and negative predictive values depend on disease prevalence and should not be assumed to transfer directly to other clinical populations. The observed profile may instead justify further investigation of the model as a high-specificity component within a validated multi-image prioritization or decision-support workflow.

The historical secondary random image-level analysis produced higher numerical image-level estimates than the primary patient-disjoint analysis. However, patient separation was not enforced in this historical dataset, and complete infant-level provenance could not be reconstructed. Within-infant dependence may therefore have contributed to optimistic performance estimates. These secondary findings are retained as exploratory methodological observations and are not interpreted as equivalent evidence of independent clinical generalization.

The raw-fundus experiment addressed whether explicit highlighting of the predicted retinal vasculature improved plus-disease classification. The VGG16 classifier trained on unmodified fundus images achieved a higher ROC-AUC than the classifier trained on U-Net-derived vessel-overlay images (0.959 vs. 0.906), together with higher accuracy, sensitivity, and specificity. Thus, the tested strategy of displaying predicted vessels in green while dimming non-vascular retinal regions did not improve classification. This result does not indicate that retinal vascular features are clinically unimportant. Rather, segmentation errors, removal or alteration of vessel appearance, background dimming, artificial contrast boundaries, and loss of contextual retinal information may have reduced the usefulness of the overlay representation.

The ophthalmologist reader study provided complementary clinical context. Ophthalmologist 1 and Ophthalmologist 2 assessed 14 of 18 and 17 of 18 plus-reference images, respectively, as Healthy. Both readers nevertheless reproduced the same severity grade for all 10 repeated images, indicating that the observed disagreement with the examination-derived reference was not explained simply by random intra-observer variability. Instead, the findings suggest that some isolated images did not contain the same diagnostic evidence as the complete multi-image examination from which their reference labels were inherited.

This distinction is central to interpretation of the classification results. Agreement between an image-level prediction and an examination-derived label does not establish that definite plus-disease features were independently visible in that particular frame or that the model relied on clinically valid vascular features. Similarly, disagreement between an ophthalmologist’s isolated-image assessment and the inherited reference label should not be interpreted as poor examination-level clinical performance. The reader findings support the clinical importance of reviewing multiple adequate views from both eyes and motivate development of models that explicitly assess image quality, select informative posterior-pole views, and combine evidence at the examination level.

Grad-CAM visualizations provided qualitative information about the spatial regions associated with selected model predictions. Activation sometimes overlapped the major vascular arcades, while selected false-negative images showed more diffuse or displaced activation. Because no localization reference standard was available and the displayed examples were selected qualitatively, these observations constitute anatomical plausibility checks only. They do not demonstrate causal model reasoning or establish that the models evaluated vascular dilation and tortuosity in the same manner as an ophthalmologist.

Overall, the study demonstrates that deep-learning models can distinguish plus-labelled from non-plus-labelled fundus images from previously unseen infants under a patient-disjoint partition. At the same time, the results identify important methodological and clinical challenges related to within-infant image correlation, examination-derived weak labels, single-image diagnostic adequacy, operating-threshold selection, and multi-image integration. These findings support continued investigation of assistive examination-level systems but do not support autonomous clinical use in their current form.

### 4.2 Clinical reference standard and unit of analysis

The distinction between the clinical reference unit and the model prediction unit is central to interpretation of this study. Plus disease is diagnosed clinically by evaluating dilation and tortuosity of the posterior-pole vessels, often using multiple views from both eyes. During neonatal fundus imaging, infant eye movement, changes in camera angle, eyelid obstruction, variable focus, uneven illumination, and incomplete retinal coverage can produce markedly different images during the same examination. One image may clearly show the optic disc and major vascular arcades, whereas another may show a peripheral field, be partially dark, or provide insufficient vascular visibility.

The clinical reference labels used in this study were derived from review of the broader multi-image examination. Individual images then inherited the corresponding plus or non-plus reference label. Consequently, a plus-labelled image should not automatically be interpreted as an image in which definite plus-disease features were independently visible. The label indicates that the image originated from an examination assigned a plus diagnosis, not necessarily that the isolated image alone met all clinical criteria for plus disease.

From a machine-learning perspective, the individual images therefore carry examination-derived weak labels rather than independent image-level annotations of visible plus-disease severity. Performance relative to these labels measures how well the model predicts the examination-derived class from an individual image. It does not establish that every correctly classified plus-labelled image independently displayed clinically definite plus-disease features. This distinction is important when interpreting both model performance and disagreement with isolated-image ophthalmologist assessment.

Patient-disjoint partitioning prevents images from the same infant from appearing in both the development and hold-out sets and therefore reduces direct infant-level leakage. However, patient-disjoint partitioning does not convert image-level predictions into patient-level predictions. A true patient- or examination-level evaluation would require a prespecified aggregation rule that combines all eligible images from an infant, eye, or examination and produces one final probability and one corresponding clinical reference diagnosis per independent case.

The primary hold-out set contained 98 images from only eight infants, and conventional image-level performance measures therefore did not account for the unequal contribution of correlated images from individual infants. Additional infant-equal-weighted, leave-one-infant-out, and stratified infant-cluster bootstrap analyses were consequently performed. These analyses demonstrated that cohort composition affected the estimated performance, particularly sensitivity. For example, DenseNet121 ROC-AUC decreased from 0.929 under conventional image weighting to 0.905 when each infant contributed equal total weight, while sensitivity decreased from 0.647 to 0.394. Leave-one-infant-out DenseNet121 ROC-AUC ranged from 0.838 to 0.989. These findings reinforce the distinction between patient-disjoint image-level evaluation and evaluation based on a large number of independent patient-level cases.

The analysis used the examination-derived reference labels available for each image. Detailed visit-level metadata were not available for all images; therefore, the labels were not retrospectively reinterpreted or reassigned across images from the same infant. All images from each infant were nevertheless retained within a single data partition, preserving patient-disjoint separation between the development and hold-out sets.

### 4.3 Within-infant prediction variability and descriptive aggregation

The exploratory infant-grouped analysis demonstrated that image-level model outputs may vary substantially among images assigned to the same infant. This was particularly evident for P01, a plus-reference infant for whom predicted plus-disease probabilities ranged from 0.015 to 0.892 and only 5 of 20 images exceeded the fixed threshold of 0.5. By contrast, P02 showed predominantly high probabilities across images from both eyes, with 28 of 30 images classified as plus disease. These different patterns indicate that the amount of diagnostically relevant information available to the classifier was not uniform across infants or across images from the same infant.

Substantial within-infant variation was observed in model-predicted probabilities. Several factors could plausibly contribute to this variation, including differences in posterior-pole coverage, camera angle, illumination, focus, eyelid obstruction, retinal field, biological inter-eye variation, and model sensitivity to acquisition-related features. However, formal image-quality, gradability, and posterior-pole adequacy annotations were not available, and the present analysis cannot determine the relative contribution of these factors. The observed probability variation should therefore not be interpreted as evidence that any specific acquisition characteristic caused the variation in model output.

The ordered image-to-image analysis provided further evidence that image selection materially influenced model output. Across 84 adjacent image pairs formed separately within 13 infant–laterality groups containing at least two images, the mean absolute change in predicted plus-disease probability was 0.142, and 11 pairs crossed the fixed threshold of 0.5. Thus, adjacent images in the recorded order for the same infant and laterality could receive substantially different probabilities and, in some cases, different binary classifications.

This effect was especially pronounced for P01, for whom seven ordered adjacent image pairs crossed the decision threshold. Consequently, the classification obtained from one available image depended strongly on which frame was selected. P02 showed greater classification stability because most of its available images remained above the decision threshold.

Importantly, the observed variability was not restricted to DenseNet121, which had originally been highlighted because of its numerical hold-out ROC-AUC. Repeating the same analysis across all five CNN architectures yielded mean adjacent probability changes ranging from 0.137 to 0.171 and threshold-crossing rates ranging from 13.1% to 21.4%. DenseNet121 was not the most variable architecture. This cross-model consistency strengthens the interpretation that dependence on image selection reflects a broader limitation of single-image classification in this dataset rather than an idiosyncrasy of one particular CNN architecture. Nevertheless, because all five analyses used the same small eight-infant hold-out cohort, these findings remain exploratory.

These results demonstrate dependence on image selection but do not establish the temporal order or timing of image acquisition. They therefore do not prove that acquiring additional images automatically improves model performance. Nevertheless, the availability of multiple views may reduce dependence on one unrepresentative frame when combined with appropriate image-quality assessment, view selection, and a prespecified aggregation method.

The benefit of multiple images depends not only on their number but also on image quality, anatomical adequacy, view selection, and the method used to combine their predictions. Additional low-quality or atypical images could introduce noise or false-positive outputs, whereas averaging all images could dilute evidence from a small number of diagnostically informative views.

Numerical differences between left- and right-eye mean probabilities were also observed for several infants. These differences could reflect genuine inter-eye asymmetry in vascular disease, which may occur clinically. However, they could also arise from unequal image numbers, differences in image quality, posterior-pole adequacy, acquisition angle, or the presence of atypical individual images. In the absence of eye-specific clinical reference labels and formal gradability assessments, the present results cannot distinguish genuine inter-eye disease asymmetry from variation in image acquisition or model behaviour.

The non-plus-reference infant P07 illustrates a potential limitation of maximum-probability aggregation. Although eight of its nine images remained below the threshold, one image received a probability slightly above 0.5. A maximum-based rule would allow this single atypical image to determine the final infant classification and would generate a false-positive result. Such an output could reflect borderline vascular appearance, an acquisition artefact, incomplete posterior-pole visualization, or another image feature that increased the model probability.

The exploratory aggregation analysis further illustrated that the resulting infant-grouped output depended on the rule used to combine image-level probabilities. At the fixed threshold of 0.5, mean and median aggregation each assigned one of the eight infants to the plus class, maximum aggregation assigned three, and averaging the three highest probabilities assigned two. These differences are presented only to illustrate sensitivity to aggregation strategy and should not be interpreted as infant- or examination-level diagnostic performance.

These findings support a clinically structured multi-stage approach to examination-level prediction. A future system could first assess image gradability and posterior-pole adequacy, exclude or down-weight non-diagnostic frames, and estimate plus-disease probabilities only for sufficiently informative views. Evidence could then be combined initially within each eye and subsequently across both eyes using a prespecified quality-weighted, top-k, or attention-based aggregation method. Representative images and uncertainty estimates could be presented alongside the final examination-level output for ophthalmologist review.

The present infant-grouped analysis remains exploratory because it included only eight infants, the number of available images differed substantially among infants, and clinical examination dates were unavailable. The analysis was therefore treated as infant-grouped rather than examination-level. The recorded image order was used only for descriptive comparison and did not provide a direct measure of elapsed time, camera position, image quality, or posterior-pole adequacy.

Any future aggregation strategy should be specified and developed using a separate development cohort and evaluated once on an independent patient- or examination-level test cohort. Prospective studies should also record image quality, anatomical field, eye laterality, acquisition sequence, and clinical examination date to determine whether quality-aware multi-image selection improves diagnostic performance compared with isolated-image classification.

### 4.4 Interpretation of the single-image ophthalmologist assessment

The reader study directly examined whether one isolated image contained sufficient visible vascular information for confident plus-disease grading. Each ophthalmologist reviewed 30 unique images without access to companion views from the same examination. Ten images were presented a second time to evaluate intra-observer repeatability.

Under the strict definition, sensitivity relative to the examination-derived reference labels was 5.6% for Ophthalmologist 1 and 0.0% for Ophthalmologist 2, while specificity was 100% for both. Under the abnormality-sensitive definition, sensitivity was 22.2% and 5.6%, respectively, with specificity remaining 100% for both. These values represent agreement between isolated-image assessments and inherited examination-derived labels, not routine examination-level ophthalmologist diagnostic accuracy.

These findings should not be interpreted as poor ophthalmologist patient-level diagnostic accuracy. The readers did not perform a standard clinical ROP examination. They were intentionally restricted to one isolated image, while the reference diagnosis had been established from the broader multi-image examination. Most disagreements occurred when images carrying a plus reference label were assessed as Healthy or Borderline/Pre-plus, which is compatible with the possibility that the selected frame did not independently show sufficient posterior-pole vascular abnormality.

Both ophthalmologists reproduced the same severity category for all 10 repeated images. Exact repeated-confidence agreement was 80% for Ophthalmologist 1 and 70% for Ophthalmologist 2, and the mean absolute confidence changes were only 0.2 and 0.3 points. The stable repeated responses argue against random reader inconsistency as the principal explanation for the apparent disagreement with the reference labels.

Many Healthy assessments were also made with high confidence. Ophthalmologist 1 assessed 14 of 18 plus-reference images as Healthy, with a mean confidence of 4.14/5, while Ophthalmologist 2 assessed 17 of 18 as Healthy, with a mean confidence of 4.59/5. These images did not merely produce uncertain responses; in isolation, they appeared non-plus to the readers. This result supports the clinical distinction between an examination-level diagnosis and the information visible in one selected frame. The reader study should not be interpreted as a substitute for systematic image-level relabelling of the complete dataset; rather, it provides a focused illustration of the potential discordance between isolated-image appearance and the examination-derived reference label.

Image quality and posterior-pole adequacy were not formally graded in the questionnaire. The study therefore cannot determine whether each disagreement resulted from insufficient field of view, blur, obstruction, illumination, subtle vascular phenotype, or another factor. Future reader studies should include explicit gradability and posterior-pole adequacy scores and, where possible, compare isolated-image grading with full multi-image examination grading by the same observers.

### 4.5 Interpretation of the exploratory secondary image-level analysis

The historical secondary analysis provided complementary information about image-level model behaviour, but its evidential role is intentionally different from that of the primary patient-disjoint analysis. Because patient identity was not enforced during partitioning and complete infant-level provenance could not be reconstructed, the secondary results may be optimistic because of within-infant dependence. They are therefore interpreted as exploratory methodological findings rather than independent clinical validation.

The secondary ResNet-18 model achieved strong image-level discrimination on the available 83-image test set. Its confusion matrix contained 45 true positives, 33 true negatives, 3 false positives, and 2 false negatives. The Brier score of 0.0573 indicated relatively low probabilistic error within this test collection, particularly for predictions near the probability extremes.

Nevertheless, the reliability diagram contained sparse intermediate probability bins with wide confidence intervals. The Brier score reflects both discrimination and calibration and should not by itself be described as proof of excellent calibration. Furthermore, calibration estimated at the image level does not establish probability reliability after predictions are aggregated across multiple images from one infant.

The training-fraction experiment suggested that image-level performance generally improved as more training images were used. However, the experiment was based on one subsampling realization, and performance was not strictly monotonic across all metrics. It therefore does not demonstrate that performance universally saturates at 80% of the available data. Repeated stratified subsampling or nested patient-wise validation would be needed to quantify variability in the learning curve.

### 4.6 Explainability and vessel-overlay findings

In selected examples, Grad-CAM activation visually overlapped portions of the major retinal vascular arcades, whereas selected false-negative examples showed weaker, more diffuse, or anatomically displaced activation. This pattern was anatomically plausible because posterior-pole vascular dilation and tortuosity define plus disease. However, Grad-CAM heatmaps are sensitive to the selected network layer, preprocessing, normalization, and visualization method. Broad activation over a vascular region does not prove that the highlighted pixels causally determined the prediction or that the network used the same reasoning as an ophthalmologist.

The raw-fundus vs. vessel-overlay experiment provided an additional assessment of vascular emphasis. The vessel-overlay images were generated from the original fundus images using binary vessel masks produced by a separately trained U-Net. Predicted vessels were highlighted in green while the remaining retinal background was dimmed. The objective was to determine whether directing the classifier toward vascular anatomy would improve plus-disease classification.

The raw-fundus VGG16 model achieved a higher ROC-AUC than the vessel-overlay model (0.959 vs. 0.906), together with higher accuracy, sensitivity, and specificity at the reported threshold. This result does not indicate that retinal vessels are unimportant. Several technical factors may explain the lower vessel-overlay performance. Segmentation errors may omit, fragment, or distort thin and low-contrast vessels. A binary mask may also reduce information about vessel calibre, colour, edge profile, local contrast, and intensity variation. Dimming the background introduces an artificial image distribution and may weaken transfer from ImageNet pretraining. In addition, the raw images retain contextual information about optic-disc location, posterior-pole visibility, vascular-arcade organization, haemorrhage, illumination, blur, and field of view.

The scientifically supported conclusion is therefore limited to the tested pipeline: highlighting predicted vessels in green while dimming the remaining retina did not improve classification. Vessel segmentation may still be useful for quantitative vascular measurements, visualization, and interpretability, but the current experiment does not establish that a vessel-overlay representation is preferable as the primary classifier input.

### 4.7 Clinical operating thresholds and potential clinical role

The fixed threshold of 0.5 was used as a reproducible operating point but was not optimized for a specific clinical use. In the primary patient-disjoint image-level analysis, DenseNet121 achieved high specificity and positive predictive value at this threshold, but sensitivity remained insufficient for a screening application. This operating profile is therefore more consistent with a potential rule-in or prioritization role than with autonomous exclusion of plus disease.

The additional development-defined high-sensitivity analysis illustrates the strong dependence of threshold-dependent performance on the intended clinical objective. For DenseNet121, lowering the operating threshold from 0.5 to the development-derived value of 0.0184 increased hold-out sensitivity from 64.7% to 98.0% and reduced false negatives from 18 to one. However, specificity decreased from 97.9% to 46.8%, and the proportion of hold-out images classified as plus increased from 34.7% to 76.5%. Similar trade-offs were observed for the other architectures, including an extreme case for EfficientNet-B0 in which 100% sensitivity was obtained only by classifying every hold-out image as plus.

These results demonstrate that threshold adjustment can substantially reduce false-negative image classifications, but high sensitivity alone does not establish a clinically useful operating point. A screening or prioritization threshold must balance missed disease against the workload generated by false-positive classifications. Furthermore, because the current analysis produces individual-image predictions, the image-level flagged fraction cannot be directly translated into examination-level referral burden. Determining a clinically appropriate threshold will require a prespecified examination-level aggregation strategy and prospective evaluation in a larger independent population.

The current findings support further investigation of an assistive multi-image workflow rather than autonomous screening. Such a system could first assess image quality and posterior-pole adequacy, estimate an image-level plus-disease probability for each gradable view, and then integrate evidence across multiple images and both eyes using a prespecified examination-level aggregation method. The resulting output could include an examination-level probability, uncertainty estimate, and representative images contributing most strongly to the prediction.

The clinical value of this workflow should be evaluated prospectively in larger, external, multicentre cohorts. Future workflow studies should determine whether the system improves case prioritization, sensitivity, reading efficiency, or inter-reader agreement without producing an unacceptable referral burden or delaying definitive ophthalmologist assessment. Future prospective studies should additionally include independently defined image-quality and posterior-pole adequacy labels and evaluate whether validated quality-based exclusion, weighting, or informative-view selection improves examination-level performance.

### 4.8 Strengths and limitations

The strengths of this study include evaluation of multiple deep-learning architectures, reporting of both threshold-dependent and threshold-independent performance measures, use of a patient-disjoint primary partition, analysis of probability reliability and model errors, and exploratory qualitative anatomical visualization using Grad-CAM. The comparison of raw-fundus and vessel-overlay inputs provided additional information about the effect of explicitly emphasizing the retinal vasculature. The ophthalmologist reader study, including repeated presentations, was particularly informative because it examined the difference between assessment of an isolated image and diagnosis based on a complete multi-image clinical examination.

The study also has important limitations. The primary hold-out set contained 98 images from only eight infants, including only three Plus-reference infants, and the number of images contributed by individual infants was highly unequal. The reported image-level observations therefore do not represent 98 independent clinical cases.

Additional cluster-aware sensitivity analyses quantified the consequences of this structure. For DenseNet121, conventional image-level ROC-AUC was 0.929, whereas infant-equal ROC-AUC was 0.905. Leave-one-infant-out ROC-AUC ranged from 0.838 to 0.989, and the exploratory 10,000-resample infant-cluster bootstrap yielded a 95% percentile interval of 0.752–0.999. The effect of weighting was particularly notable for sensitivity, which decreased from 0.647 under conventional image weighting to 0.394 when each infant contributed equal total weight. Comparable cohort dependence was observed for the other evaluated architectures.

These analyses quantify, but do not eliminate, the limitation imposed by the small number of independent infant clusters. In particular, the wide cluster-bootstrap intervals demonstrate that the primary point estimates should not be regarded as precise or stable estimates of population-level clinical performance. Patient-disjoint partitioning prevents direct infant overlap between model development and hold-out evaluation, but it does not substitute for validation in a substantially larger number of independent patients. Larger external, multicentre, device-diverse, and prospectively collected cohorts are therefore required before patient- or examination-level diagnostic performance can be established.

The evaluated architecture panel consisted of established convolutional backbones and did not include more recent architectures such as ConvNeXt or vision-transformer-based models. Accordingly, the present study should not be interpreted as a state-of-the-art architecture benchmark. The central findings concern the evaluation framework, patient-disjoint partitioning, examination-derived weak image labels, and within-infant variability rather than superiority of a particular backbone. Future studies using larger independent patient-level cohorts should evaluate whether these findings remain consistent with more recent vision architectures.

Clinical and acquisition metadata were incomplete in this retrospective dataset. Gestational age, birth weight, postmenstrual age at imaging, detailed ROP zone and stage, pre-plus status, prior treatment, eye-specific clinical diagnosis, verified examination date, and operator information were not consistently available. Consequently, clinical spectrum balance between the development and hold-out cohorts and performance stratified by these variables could not be evaluated. Formal image-quality, gradability, and posterior-pole adequacy scores were also unavailable.

Although automated preprocessing removed black background borders, the present study did not formally exclude shortcut learning from illumination, field of view, acquisition angle, image quality, camera-related characteristics, or other non-diagnostic image features. Model agreement with the examination-derived reference label should therefore not be interpreted as evidence that the classifier relied specifically or exclusively on clinically valid vascular dilation and tortuosity.

The secondary dataset was divided at the image level without enforced patient separation. Images from the same infant could therefore occur in both development and test partitions, potentially producing optimistic performance estimates. The secondary findings should be interpreted as exploratory image-level results and not as independent patient-level generalization.

The ophthalmologist reader study included only two ophthalmologists and 30 unique images. Image quality, gradability, and posterior-pole adequacy were not formally scored. In addition, the clinical reference labels were derived from the complete multi-image examination rather than from independent grading of the visible disease severity in each isolated image. Consequently, the reported image-level performance quantifies prediction of the examination-derived class from individual photographs rather than diagnostic accuracy against an independently established image-level reference standard. The reader findings should therefore not be interpreted as estimates of routine ophthalmologist examination-level diagnostic accuracy.

Finally, the Grad-CAM analysis was exploratory and qualitative. No ophthalmologist-defined localization masks or regions of interest were available, precluding numerical assessment of spatial agreement between the model explanation maps and clinically relevant retinal regions. Grad-CAM also has limited spatial resolution and should not be interpreted as a precise localization method or as evidence of causal feature use. Nevertheless, the visualizations were retained as qualitative anatomical plausibility aids for selected model predictions. The complementary LIME analysis was subject to the same limitation: in the absence of ophthalmologist-defined localization masks, its highlighted regions could not be quantitatively validated as clinically correct anatomical localization.

The vessel-overlay experiment evaluated one particular segmentation and visualization strategy and does not establish the general diagnostic value of vessel-based representations. Larger, external, multicentre, device-diverse, and prospectively collected cohorts are required before patient- or examination-level clinical performance can be established.

## 5 Conclusion

This study provides a patient-disjoint image-level evaluation of deep-learning models for ROP plus-disease classification in real-world neonatal fundus images. Restricting all images from each infant to a single data partition prevented direct infant overlap between model development and hold-out evaluation. DenseNet121 achieved a conventional image-level ROC-AUC of 0.929 and specificity of 97.9% at the fixed threshold of 0.5. However, cluster-aware sensitivity analyses demonstrated dependence on the composition of the eight-infant hold-out cohort: infant-equal-weighted image-level ROC-AUC was 0.905 and leave-one-infant-out ROC-AUC ranged from 0.838 to 0.989. These findings support interpretation of the results as exploratory patient-disjoint image-level evidence rather than as precise estimates of clinical diagnostic performance.

Substantial variation in predicted probabilities was observed among images assigned to the same infant and across all five evaluated CNN architectures. Because complete examination identifiers were unavailable, these observations were treated descriptively and were not converted into infant- or examination-level diagnostic performance. Future multi-image systems should use verified eye and examination identifiers together with prespecified aggregation strategies developed and validated at the appropriate clinical unit.

The historical secondary random image-level analysis provided complementary exploratory information regarding architecture behaviour, training-data sensitivity, probability reliability, model visualization, and vessel-overlay representations. Because patient separation was not enforced and complete infant-level provenance could not be reconstructed retrospectively, these findings are interpreted only as supportive image-level analyses and not as evidence of independent clinical generalization.

The ophthalmologist reader study provided additional clinical context for the weak image-level reference labels. Two ophthalmologists independently assessed 30 unique isolated fundus images, and 10 of these images were presented a second time to assess intra-observer repeatability. Exact three-category inter-observer agreement was 26/30 (86.7%), and both ophthalmologists reproduced the same severity grade for all 10 repeated images. Nevertheless, 14/18 and 17/18 plus-reference images were assessed as Healthy by Ophthalmologists 1 and 2, respectively. Together, these findings indicate that examination-derived labels inherited by individual images are not equivalent to independent image-level annotations of visible plus-disease severity. High repeatability of isolated-image grading therefore does not imply agreement with a reference diagnosis established from a broader multi-image examination.

Overall, the findings support development of assistive examination-level systems rather than autonomous image-level screening. Future systems should assess image gradability and posterior-pole adequacy, select diagnostically informative views, integrate evidence within each eye and across both eyes using prespecified aggregation rules, and communicate prediction uncertainty. Such approaches should be developed and evaluated using larger, external, multicentre, and prospectively collected cohorts with clinically selected operating thresholds and independent patient- or examination-level reference standards before clinical implementation is considered.

## Data Availability

The datasets presented in this article are not readily available because The clinical retinal images are not publicly available because they are subject to patient-privacy requirements, institutional data-governance conditions, and commercial-use restrictions. The images were acquired and used for research under approval of the Ethics Committee of University Hospital Ostrava (reference no. 544/2018). The authors are not authorized to publicly redistribute the clinical images. Source code and analysis scripts may be made available by the corresponding author upon reasonable request, subject to institutional approval and applicable usage conditions. Requests to access the datasets should be directed to Rouhollah Kian Ara rouhollah.kian.ara@vsb.cz. Source code for image preprocessing, model training and inference, patient-disjoint data partitioning, grouped cross-validation, hold-out evaluation, development-set-defined operating-point analysis, within-infant variability analysis, cluster-aware statistical analysis, and generation of the reported analytical outputs is publicly available at: https://github.com/kaushikGeom/ROP_Classification. The repository includes the model and analysis configurations, preprocessing procedures, random seed handling, and scripts required to reproduce the reported methodology and statistical analyses when used with the corresponding clinical data and derived model outputs. The original clinical images are not included because of the restrictions described above.
