# Supplementary material for: Explainability-informed patient-disjoint evaluation of deep learning for plus disease in retinopathy of prematurity with an ophthalmologist reader study
Source: Front Med (Lausanne). 2026 Sep 30;13:1960317. doi: 10.3389/fmed.2026.1960317 (PMC13661440; doi:10.3389/fmed.2026.1960317)
Supplement: Supplementary file 1 [file Datasheet1.pdf]

Supplementary Material

S1 Supplementary Methods

S1.1 Image Selection and Presentation Sequence

Thirty unique isolated fundus images were selected by computer-generated random sampling from the eligible images in the secondary held-out image-level test set. Image selection was completed before the reader assessment, and neither ophthalmologist participated in or was informed about the sampling procedure. The selected set comprised 18 images carrying a plus examination-derived reference label and 12 carrying a non-plus examination-derived reference label.

Ten of the 30 unique images were selected for repeat presentation, resulting in 40 image presentations in total. The repeated images were inserted into the assessment sequence without any indication that they had been presented previously. The complete presentation sequence was randomized, and no information distinguished the first presentation from the repeated presentation. Consequently, the ophthalmologists were unaware of which images were repeated and of the positions at which repeated images appeared.

The 30 unique images were used for reader-reference and inter-observer analyses. The 10 repeated presentations were used only to assess intra-observer repeatability.

S1.1.1 Reader Blinding and Independent Assessment

Two ophthalmologists with significant experience in paediatric retinal imaging or ROP assessment independently assessed the images.

The ophthalmologists were blinded to the image-selection procedure, examination-derived reference labels, model predictions, the other ophthalmologist's assessments, and the identity of the repeated images. They were also blinded to infant identity and to the other images belonging to the same infant or clinical examination. Therefore, each assessment was based exclusively on the vascular appearance visible in one isolated fundus image.

No information from the complete clinical examination was provided, including the reference diagnosis derived from multiple images, the assessment of the fellow eye, or the longitudinal clinical record. This design was intended to evaluate isolated-image interpretation rather than routine examination-level diagnostic performance.

For each presentation, the ophthalmologists independently assigned one of three ordered categories:

1. Healthy;
2. Borderline/Pre-plus; or
3. Plus.

S1.1.2 Image Display Conditions

Images were presented individually through Google Forms. The ophthalmologists completed the assessment independently using their own display devices. Display size, resolution, calibration,

browser magnification, and ambient lighting were not standardized or recorded. No model prediction, Grad-CAM map, vessel segmentation, reference label, or other computer-generated annotation was shown.

S1.1.3 Provenance of Model Predictions

The model predictions used in the reader comparison were generated by the ResNet-18 classifier from the secondary random image-level analysis. The reader-study images belonged to the held-out test set and were not used for model fitting or checkpoint selection.

For each image, the five fold-specific ResNet-18 models independently generated softmax probabilities for the plus and non-plus classes. The class probabilities were averaged across the five models to obtain the fold-ensemble prediction:

$$\bar{p}_{\text{plus}} = \frac{1}{5}\sum_{k=1}^{5} p_{\text{plus}}^{(k)}, \tag{S1}$$

where $p_{\text{plus}}^{(k)}$ denotes the plus-class probability produced by fold-specific model k. An image was classified as plus when

$$\bar{p}_{\text{plus}} \geq 0.5, \tag{S2}$$

and as non-plus otherwise.

Model predictions were generated independently of the ophthalmologist assessments and were not available to either ophthalmologist during image grading. Similarly, the model had no access to the ophthalmologist assessments, confidence scores, or repeat-presentation status.

S1.1.4 Reference Labels and Analytical Definitions

The binary reference labels were derived from the corresponding complete clinical examinations and were inherited by the individual images. The reference assessment could therefore incorporate multiple retinal views and information from both eyes when available, which were not available to the ophthalmologists during isolated-image grading.

For the strict reader analysis, only a definite Plus assessment was treated as positive, while Healthy and Borderline/Pre-plus were treated as negative. For the abnormality-sensitive analysis, both Borderline/Pre-plus and Plus were treated as positive, while Healthy was treated as negative.

This comparison consequently measured agreement between isolated-image assessments and examination-derived reference labels. It was not designed to estimate the routine examination-level diagnostic accuracy of the participating ophthalmologists.

S1.2 Grad-CAM Generation and Visualization

Gradient-weighted Class Activation Mapping (Grad-CAM) was used to qualitatively visualize image regions associated with the ResNet-18 classification outputs [1]. Grad-CAM was applied to the five fold-specific ResNet-18 checkpoints retained during the secondary random image-level analysis.

Each fundus image was converted to RGB, resized to 224×224 pixels, transformed to a tensor, and normalized using the ImageNet channel means $(0.485, 0.456, 0.406)$ and standard deviations $(0.229, 0.224, 0.225)$. No stochastic augmentation was applied during Grad-CAM generation.

The final convolutional block of ResNet-18, `layer4`, was selected as the target layer. For a target class c, the class-specific logit y^c was backpropagated through the network. Let A_{ij}^k denote the activation at spatial position (i, j) in feature map k. The importance weight for feature map k was calculated by spatially averaging the gradients:

$$\alpha_k^c = \frac{1}{Z} \sum_i \sum_j \frac{\partial y^c}{\partial A_{ij}^k}, \tag{S3}$$

where Z denotes the number of spatial locations in the feature map. The class-specific Grad-CAM map was then calculated as

$$L_{\text{Grad-CAM}}^c = \text{ReLU}\left(\sum_k \alpha_k^c A^k\right). \tag{S4}$$

The ReLU operation retained activation associated positively with the target class. Each resulting map was min–max normalized to the interval $[0, 1]$.

A separate Grad-CAM map was generated from each of the five fold-specific models. The five normalized maps were averaged element-wise, and the ensemble map was subsequently renormalized to $[0, 1]$. Thus, the visualization represented the mean spatial activation pattern across the five fold-specific models rather than the activation from a single checkpoint.

To reduce activation over the black image background, a retinal region-of-interest mask was generated from the original RGB image. The mean intensity across the three colour channels was calculated for every pixel, and pixels with a mean intensity greater than 20 were assigned to the retinal region. The binary mask was resized to the Grad-CAM resolution using bilinear interpolation, multiplied by the ensemble Grad-CAM map, and the masked map was renormalized. When masking produced an effectively empty map, the unmasked ensemble Grad-CAM map was retained.

For visualization, the masked Grad-CAM map was resized to the original image dimensions using bilinear interpolation. A jet colour map was applied, and the heatmap was blended with the original RGB fundus image using an opacity of 0.45:

$$I_{\text{overlay}} = 0.45 I_{\text{heatmap}} + 0.55 I_{\text{fundus}}. \tag{S5}$$

For each image, class-specific maps were generated for the plus class ($c = 1$) and the non-plus class ($c = 0$). An additional displayed map corresponded to the predicted class. The predicted class was determined from the mean plus-disease probability produced by the five fold-specific models using the fixed threshold of 0.5.

The Grad-CAM maps were inspected qualitatively to determine whether model activation overlapped anatomically relevant retinal regions, particularly the optic-disc region and major vascular arcades. No pixel-level localization reference standard was available. Consequently, the maps were interpreted only as qualitative anatomical plausibility visualizations and not as evidence of causal feature use, diagnostic validity, or reasoning equivalent to that of an ophthalmologist.

S2 Supplementary Tables

Table S1: **Patient-disjoint partition of the primary dataset.**

Partition	Infants	Images	Plus-labelled images	Non-plus-labelled images
Development pool	30	634	331	303
Patient-disjoint hold-out set	8	98	51	47
Total	38	732	382	350

Note: Label counts refer to individual images. All images associated with the same infant were assigned to one partition. Predictions and performance measures were calculated at the image level.

Table S2: Descriptive post hoc infant-grouped aggregation of image-level DenseNet121 probabilities in the primary patient-disjoint hold-out cohort. The same 98 image-level predictions from eight infants were combined using four alternative aggregation rules. These analyses were exploratory and were not interpreted as validated infant- or examination-level diagnostic performance.

Aggregation rule	Infants assigned plus	Infants assigned non-plus
Mean probability	1/8	7/8
Median probability	1/8	7/8
Maximum probability	3/8	5/8
Mean of three highest probabilities	2/8	6/8

Note: Aggregated probabilities were converted to descriptive binary outputs using the fixed threshold of 0.5. The table is intended only to demonstrate how different post hoc probability-combination rules can produce different outputs from the same underlying image-level predictions. Because the cohort contained only eight infants and complete examination identifiers were unavailable, no infant- or examination-level diagnostic performance is inferred from these results.

S2.1 Patient-Disjoint Image-Level Training-Fraction Analysis

An exploratory training-fraction analysis was performed using ResNet-18 within the primary patient-disjoint framework. The same independent 98-image hold-out set from eight infants was used for evaluation at each training-data fraction.

At the fixed threshold of 0.5, sensitivity and F1-score were numerically highest when the complete training data were used, whereas ROC-AUC and PR-AUC were numerically highest at the 60% training fraction (Supplementary Table S3). Performance was not monotonic across all metrics. These findings therefore do not establish a general learning-curve saturation point or demonstrate that a particular training fraction is optimal.

The analysis was based on one subsampling realization at each fraction. Repeated patient-wise subsampling would be required to estimate variability associated with training-set composition.

Table S3: **Exploratory ResNet-18 image-level performance across training-data fractions in the primary patient-disjoint analysis.**

Training-data fraction	Accuracy	Sensitivity	Specificity	F1-score	ROC-AUC	PR-AUC	Brier score
20%	0.612	0.314	0.936	0.457	0.847	0.857	0.216
40%	0.724	0.569	0.894	0.682	0.874	0.883	0.169
60%	0.724	0.490	**0.979**	0.649	**0.893**	**0.914**	0.182
80%	0.714	0.490	0.957	0.641	0.890	0.901	0.183
100%	**0.786**	**0.667**	0.915	**0.764**	0.869	0.889	**0.164**

Note: All fractions were evaluated on the same patient-disjoint hold-out set comprising 98 images from eight infants. Metrics were calculated at the image level using the fixed threshold of 0.5. Bold values indicate the numerical maximum, or minimum for the Brier score, and do not establish statistical superiority. The analysis used one subsampling realization at each fraction and should be interpreted as exploratory.

Table S4: Exploratory ResNet-18 image-level ROC-AUC across training-data fractions in the secondary random image-level analysis.

Training-data fraction	ROC-AUC
20%	0.900
40%	0.912
60%	0.946
80%	**0.975**
100%	0.974

Note: All fractions were evaluated on the same 83-image historical secondary test set. ROC-AUC is threshold-independent. One subsampling realization was evaluated at each fraction, and the analysis should therefore be interpreted as exploratory.

Table S5: Cluster-aware sensitivity analyses for the primary patient-disjoint hold-out cohort. Conventional image-level metrics are compared with infant-equal-weighted metrics, for which each infant contributed equal total weight irrespective of the number of available images. The leave-one-infant-out (LOIO) range summarizes ROC-AUC after successive exclusion of each infant. Exploratory 95% percentile intervals were obtained from 10,000 stratified infant-cluster bootstrap resamples. These analyses characterize sensitivity to cohort composition and should not be interpreted as validated patient-level diagnostic performance.

**Model**	**Image-level AUC**	**Infant-equal AUC**	**Image Sens.**	**Infant-equal Sens.**	**Image Spec.**	**Infant-equal Spec.**	**LOIO AUC range**	**Max $\lvert\Delta AUC\rvert$**	**Cluster-bootstrap AUC 95% interval**
EfficientNet-B0	0.861	0.800	0.549	0.356	0.936	0.933	0.804–0.914	0.057	0.668–0.944
DenseNet121	**0.929**	**0.905**	0.647	0.394	0.979	0.978	0.838–0.989	0.091	0.752–0.999
ResNet-50	0.904	0.899	0.647	0.406	0.979	0.978	0.797–0.978	0.107	0.701–0.997
MobileNetV3 Large	0.927	0.920	0.627	0.389	1.000	1.000	0.830–0.994	0.098	0.769–0.999
ResNet-18	0.869	0.787	0.667	0.411	0.915	0.913	0.721–0.962	0.147	0.588–0.991

Table S6: Cross-architecture analysis of within-infant variability in the primary patient-disjoint hold-out cohort. Adjacent available images were paired only within the same infant and laterality group after ordering by recorded sequence index. The sequence index was used solely for descriptive organization and was not interpreted as verified acquisition timing. Threshold crossings indicate adjacent image pairs whose predicted class differed at the fixed threshold of 0.5.

**Model**	**Adjacent pairs**	**Mean $\lvert\Delta\rvert$**	**Median $\lvert\Delta\rvert$**	**Max $\lvert\Delta\rvert$**	**Threshold crossings**	**Crossing fraction**	**Infants with crossings**	**Mean infant SD**
EfficientNet-B0	84	0.171	0.134	0.783	18	21.4%	3	0.164
DenseNet121	84	0.142	0.087	0.708	11	13.1%	3	0.121
ResNet-50	84	0.148	0.080	0.883	18	21.4%	3	0.142
MobileNetV3 Large	84	0.163	0.095	0.736	12	14.3%	2	0.139
ResNet-18	84	0.137	0.082	0.668	11	13.1%	4	0.168

Table S7: Development-defined high-sensitivity operating points and performance on the independent patient-disjoint hold-out set. Thresholds were selected exclusively from development out-of-fold predictions to achieve sensitivity of at least 90%, while maximizing specificity. "Plus" denotes the percentage of hold-out images classified as plus and should not be interpreted as an examination-level referral rate.

Model	Thr.	Dev. Se.	Dev. Sp.	Test Se.	Test Sp.	FN	FP	Plus (%)
EfficientNet-B0	0.0051	90.0	27.7	100.0	0.0	0	47	100.0
DenseNet121	0.0184	90.0	48.5	98.0	46.8	1	25	76.5
ResNet-50	0.0157	90.0	48.8	96.1	42.6	2	27	77.6
MobileNetV3 Large	0.0267	90.0	49.5	100.0	34.0	0	31	83.7
ResNet-18	0.0586	90.0	52.1	94.1	38.3	3	29	78.6

S3 Supplementary Figures

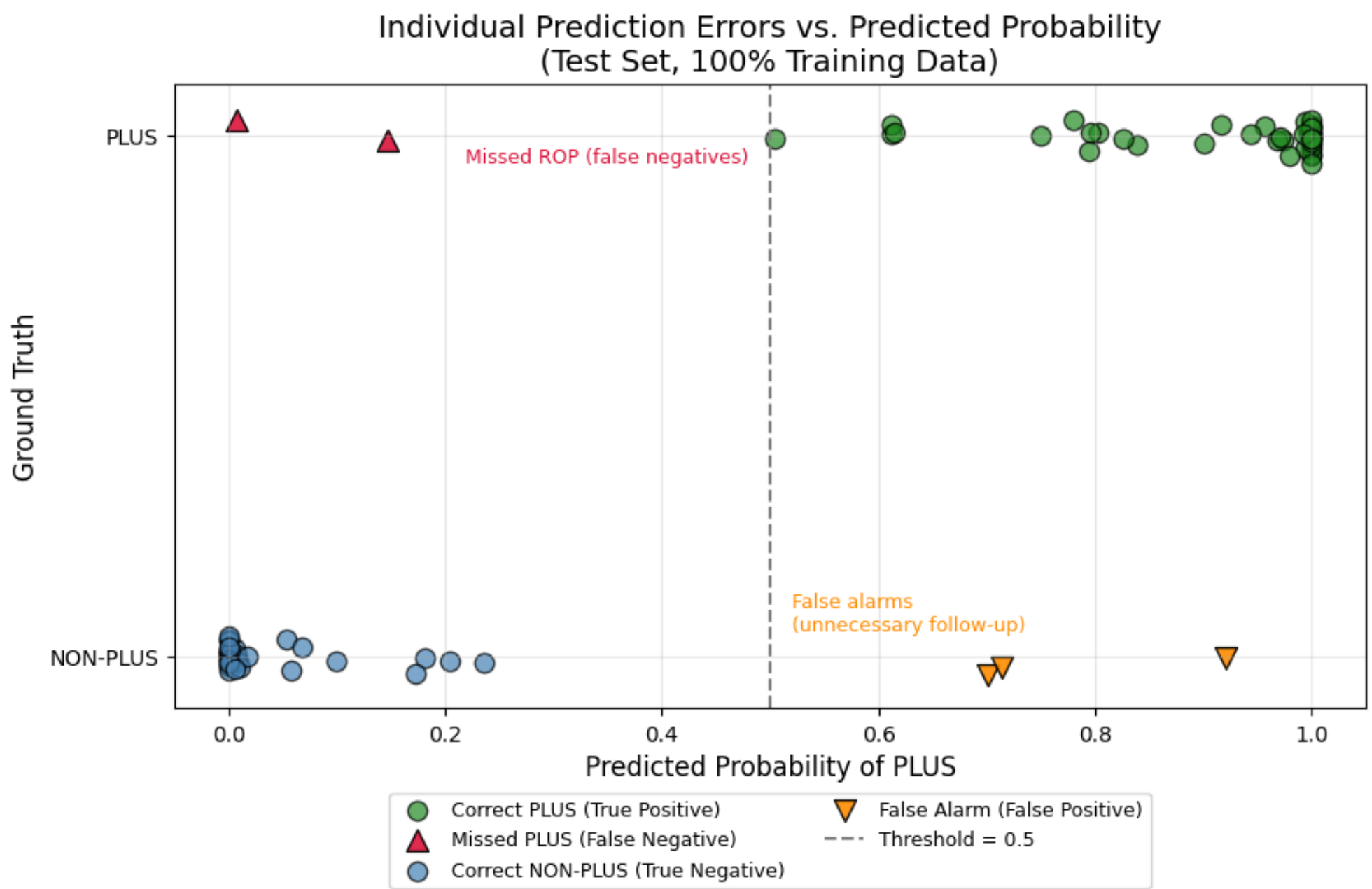

Figure S1: **Image-level predicted plus-disease probabilities on the secondary 83-image test set.** The test set comprised 47 plus-reference and 36 non-plus-reference images. The vertical dashed line indicates the fixed decision threshold of 0.5. Red triangles identify the two false-negative plus-reference images, and orange inverted triangles identify the three false-positive non-plus-reference images. Predictions were generated by the ResNet-18 fold ensemble. Reference classes were derived from the corresponding clinical examinations and inherited by the individual images.

The following figures provide the ROC curves and confusion matrices for the exploratory image-level comparison between the raw-fundus and vessel-overlay VGG16 pipelines. Both pipelines were evaluated using the same 83-image test set, comprising 47 plus-reference and 36 non-plus-reference images.

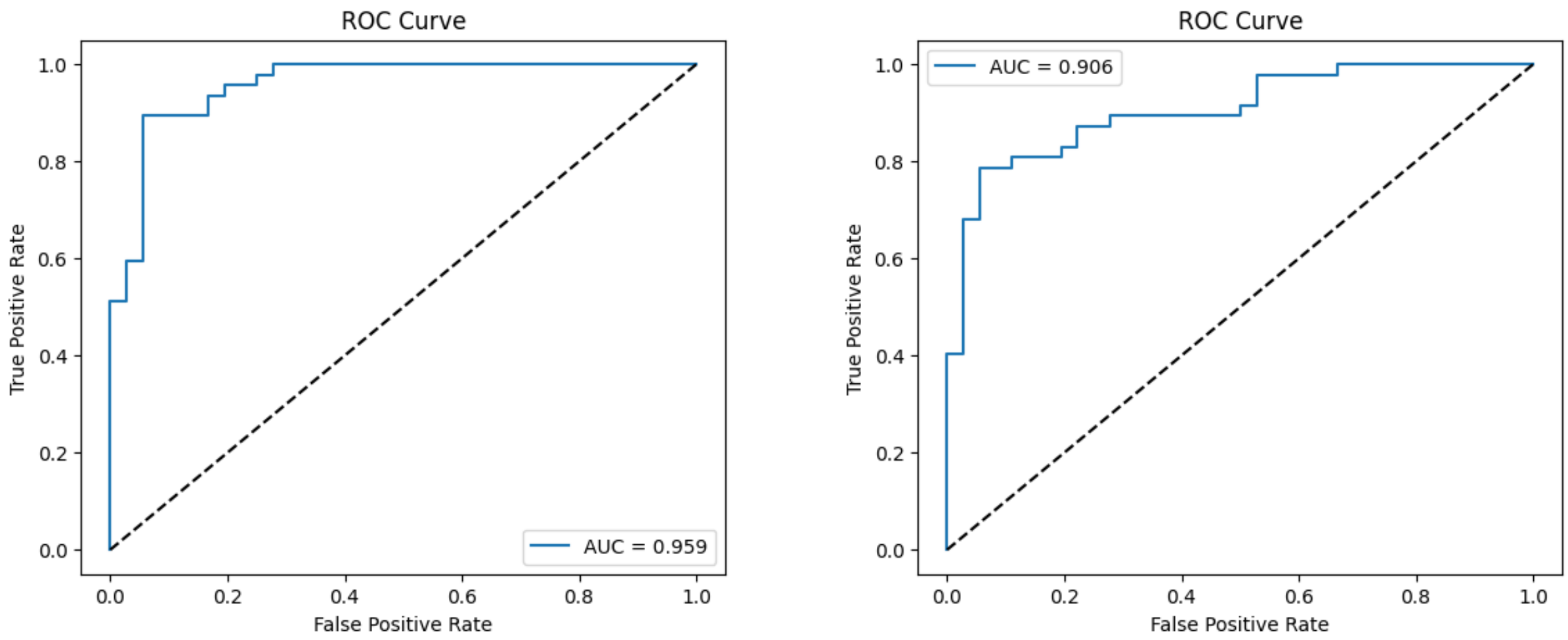

(a) Raw-fundus pipeline; ROC-AUC = 0.959.

(b) Vessel-overlay pipeline; ROC-AUC = 0.906.

Figure S2: **ROC curves for the raw-fundus and vessel-overlay VGG16 pipelines.** Both models were evaluated on the same 83-image test set. The raw-fundus pipeline produced a numerically higher ROC-AUC than the vessel-overlay pipeline. No paired inferential comparison was performed, and the difference should therefore be interpreted descriptively.

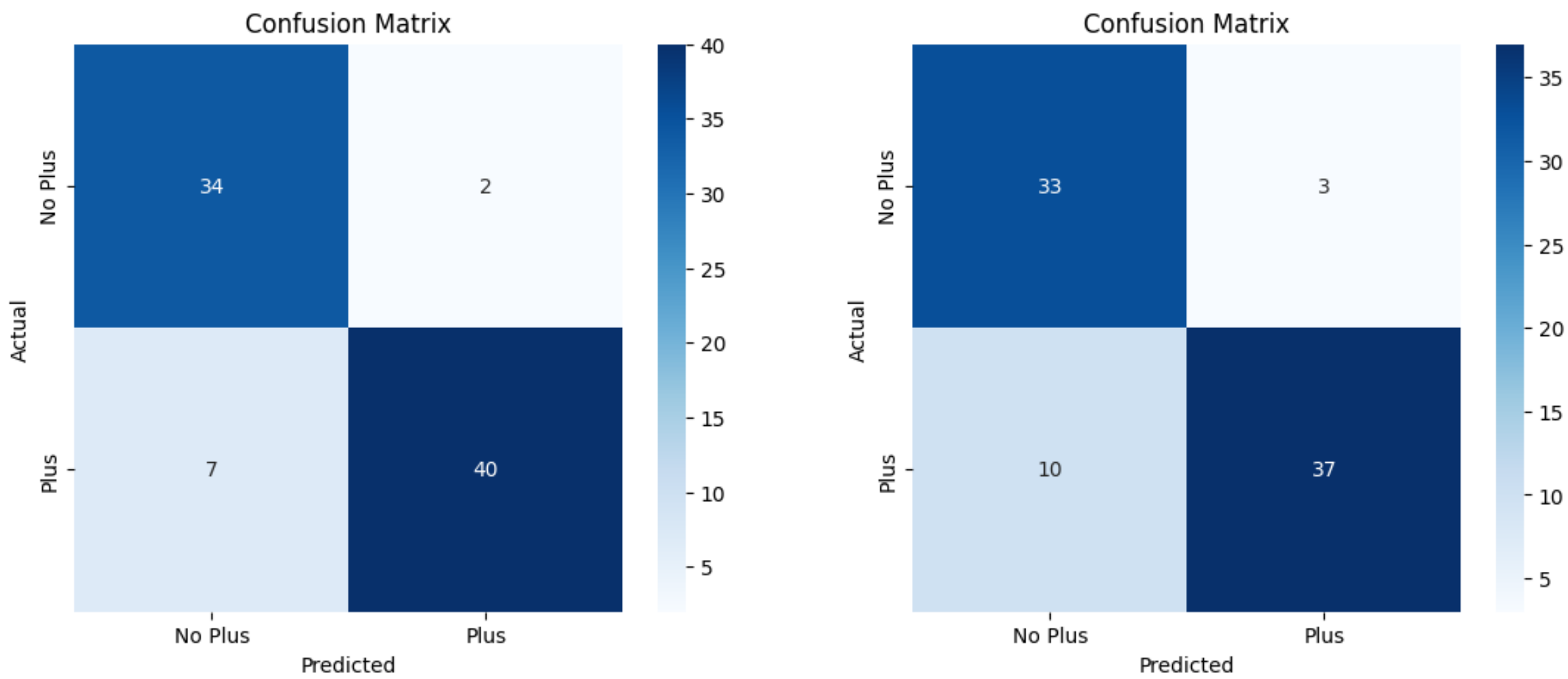

(a) Raw-fundus pipeline.

(b) Vessel-overlay pipeline.

Figure S3: **Confusion matrices for the raw-fundus and vessel-overlay VGG16 pipelines.** The raw-fundus model produced 34 true-negative, 2 false-positive, 7 false-negative, and 40 true-positive predictions. The vessel-overlay model produced 33 true-negative, 3 false-positive, 10 false-negative, and 37 true-positive predictions. Plus disease was treated as the positive class.

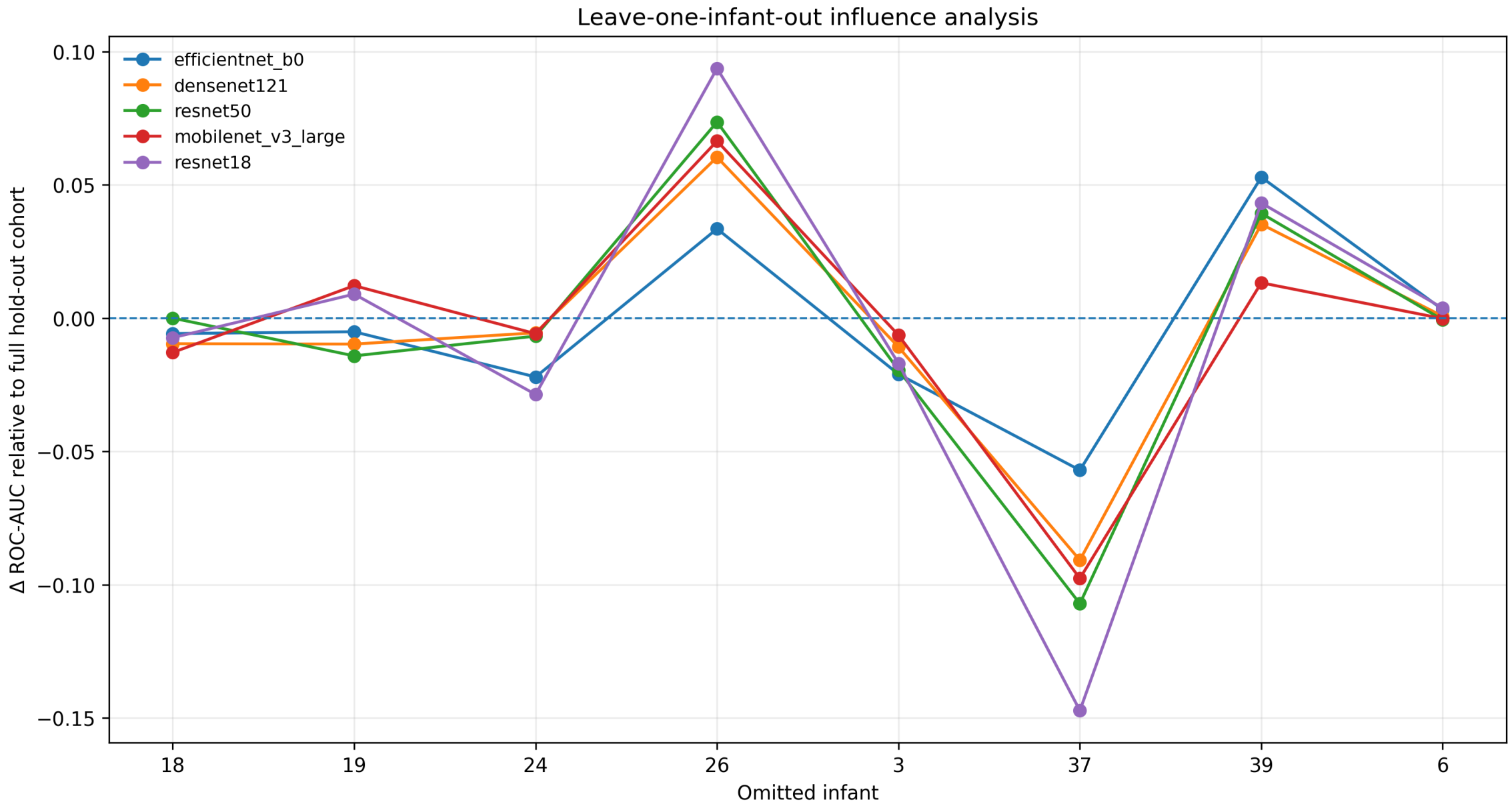

Figure S4: Leave-one-infant-out influence analysis for the primary patient-disjoint hold-out cohort. The figure shows the change in ROC-AUC after exclusion of all images associated with one infant, relative to the corresponding ROC-AUC calculated using the complete eight-infant hold-out cohort. Positive values indicate an increase in ROC-AUC following omission, whereas negative values indicate a decrease. The five evaluated CNN architectures showed their largest changes following omission of heavily represented plus-reference infants, demonstrating sensitivity of image-level performance estimates to the composition of the small hold-out cohort. Models were not retrained during this analysis; the original fixed hold-out predictions were retained.

Supplementary References

[1] R. R. Selvaraju, M. Cogswell, A. Das, R. Vedantam, D. Parikh, and D. Batra. Grad-CAM: Visual explanations from deep networks via gradient-based localization. In *Proceedings of the IEEE International Conference on Computer Vision*, pages 618–626, 2017. doi: 10.1109/ICCV.2017.74.
