# Supplementary material for: Explainability-informed patient-disjoint evaluation of deep learning for plus disease in retinopathy of prematurity with an ophthalmologist reader study
Source: Front Med (Lausanne). 2026 Sep 30;13:1960317. doi: 10.3389/fmed.2026.1960317 (PMC13661440; doi:10.3389/fmed.2026.1960317)
Supplement: Supplementary file 2 [file Table1.docx]

**CLAIM 2024 Reporting Checklist**

**Explainability-Informed Patient-Disjoint Evaluation of Deep Learning for Plus Disease in Retinopathy of Prematurity with an Ophthalmologist Reader Study**

Completed against the final uploaded main manuscript (43 pages) and final Supplementary Material (9 pages).

Completion note. Every checklist item is answered. “Yes” indicates that the requested reporting element is addressed in the manuscript or Supplement, including transparent reporting that an analysis was not performed when that disclosure satisfies the reporting criterion. “No” is reserved for information that is genuinely absent or not recoverable, and “N/A” for items that are not applicable. No unavailable information has been inferred or reconstructed.

**Guideline:** Tejani AS et al. Checklist for Artificial Intelligence in Medical Imaging (CLAIM): 2024 Update. Radiology: Artificial Intelligence. 2024;6(4):e240300. DOI: 10.1148/ryai.240300.

| **Item** | **Reporting criterion** | **Status** | **Section / locator reference** | **Completed response / explanation** |
| --- | --- | --- | --- | --- |
| **1** | Identification as an AI/deep-learning study | Yes | Abstract | The title identifies deep learning and the structured abstract reports evaluation of five pretrained CNNs, patient-disjoint evaluation, key performance measures, and the ophthalmologist reader study. |
| **2** | Structured abstract | Yes | Abstract | The abstract is structured as Background, Methods, Results, and Conclusions and reports the primary dataset, patient-disjoint hold-out, principal performance findings, reader study, and limitations. |
| **3** | Clinical/scientific background and intended clinical context | Yes | Introduction; Discussion - 4.7 Clinical Operating Thresholds and Potential Clinical Role; 5 Conclusion | The manuscript describes ROP plus-disease assessment, multi-image clinical review, limitations of isolated images, prior AI work, and a potential assistive/prioritization role rather than autonomous screening. |
| **4** | Study objectives/hypotheses | Yes | Introduction - Study objectives | Six explicit study objectives are listed, covering patient-disjoint performance, within-infant variability, exploratory secondary analyses, vessel overlays, ophthalmologist assessment, and qualitative explainability. |
| **5** | Prospective or retrospective study design | Yes | Materials and Methods - 2.1 Ethics Approval; 2.4 Study Design and Analytical Hierarchy | The study is explicitly retrospective; ethics approval, study setting, and the analytical hierarchy are reported. |
| **6** | Study goal and intended classification task | Yes | Introduction - Study objectives; Materials and Methods - 2.4 Study Design and Analytical Hierarchy | The principal task is binary image-level classification of plus-labelled versus non-plus-labelled fundus images, with partitioning unit and prediction unit explicitly distinguished. |
| **7** | Data sources | Yes | Materials and Methods - 2.2 Dataset and Clinical Reference Standard; Statements - Data Availability Statement | The institutional Phoenix ICON image collections, primary patient-disjoint dataset, historical secondary dataset, and data-availability restrictions are described. |
| **8** | Eligibility criteria and sampling frame | No | Materials and Methods - 2.2.1 Primary Patient-Disjoint Image-Level Dataset; 2.2.2 Historical Secondary Image-Level Dataset; 2.5 Model Architectures, Training, and Inference | The analyzed cohorts and absence of post-hoc subjective quality exclusion are reported, but a complete participant/image eligibility framework, acquisition period, and source-series sampling frame are not reported. |
| **9** | Image preprocessing | Yes | Materials and Methods - 2.5 Model Architectures, Training, and Inference | Automated foreground cropping, resizing to 224×224 pixels, ImageNet normalization, and training augmentation are specified; no augmentation was applied during validation or final testing. |
| **10** | Selection/cropping of image data | Yes | Materials and Methods - 2.5 Model Architectures, Training, and Inference | The automated black-border cropping algorithm, mean-intensity threshold, bounding box, square crop, 10-pixel margin, and resizing are described. |
| **11** | De-identification | Yes | Materials and Methods - 2.1 Ethics Approval; Statements - Ethics Statement | Images were de-identified before transfer/research analysis and handled in accordance with institutional data-governance requirements. |
| **12** | Missing data handling | Yes | Materials and Methods - 2.2 Dataset and Clinical Reference Standard; Discussion - 4.8 Strengths and Limitations | Clinical and acquisition metadata that could not be reliably linked across the classification cohort are explicitly listed and were not retrospectively inferred or imputed. |
| **13** | Image acquisition protocol/device | Yes | Materials and Methods - 2.2 Dataset and Clinical Reference Standard | All retinal images were acquired using a Phoenix ICON wide-field retinal imaging system at native resolution 1240×1240 pixels; additional acquisition variables were not consistently linkable across the retrospective cohort. |
| **14** | Reference-standard construction | Yes | Materials and Methods - 2.2 Dataset and Clinical Reference Standard | Binary plus/non-plus clinical reference labels were assigned by one of two experienced ROP ophthalmologists (J.T. or K.M.) after review of the complete available fundus-image set for the corresponding clinical case; each infant was assessed by one ophthalmologist. |
| **15** | Rationale and limitations of the reference standard | Yes | Materials and Methods - 2.2 Dataset and Clinical Reference Standard; Discussion - 4.2 Clinical Reference Standard and Unit of Analysis; 4.8 Strengths and Limitations | The manuscript explains that the available reference is clinical image-set/examination-derived rather than independent image-level severity grading, that individual images inherit the clinical label, and that the resulting labels constitute weak image-level supervision. These limitations are explicitly discussed. |
| **16** | Source and qualifications of reference annotations | Yes | Materials and Methods - 2.2 Dataset and Clinical Reference Standard | Reference labels were assigned by one of two experienced ROP ophthalmologists (J.T. and K.M.). The grader identity for individual retrospective cases was not retained, and no separate multi-grader consensus/adjudication procedure was used. |
| **17** | Test-set annotation/reference procedure | Yes | Materials and Methods - 2.2 Dataset and Clinical Reference Standard; 2.2.1 Primary Patient-Disjoint Image-Level Dataset; 2.2.2 Historical Secondary Image-Level Dataset | Analyzed images inherited the available clinical image-set/examination-derived binary reference labels. The same reference-label interpretation is used for the primary and historical secondary analyses. |
| **18** | Inter-/intrarater variability of the original reference standard | N/A | Materials and Methods - 2.2 Dataset and Clinical Reference Standard; Results - 3.7 Single-Image Ophthalmologist Reader Study | Each infant’s original binary clinical reference was assigned by a single ophthalmologist and no multi-grader consensus/adjudication was used; therefore inter-rater variability of the original reference standard is unavailable. The separate reader study evaluates isolated-image reader agreement and repeatability and is not treated as validation of the original clinical reference. |
| **19** | Assignment to training/validation/test partitions | Yes | Materials and Methods - 2.2.1 Primary Patient-Disjoint Image-Level Dataset; 2.2.2 Historical Secondary Image-Level Dataset; 2.5.2 Primary Patient-Disjoint Model Development; 2.5.3 Secondary Random Image-Level Model Development | The primary infant-disjoint split, grouped five-fold cross-validation, independent 98-image hold-out, historical secondary image-level split, and secondary five-fold stratified cross-validation are described. |
| **20** | Level at which data partitions are disjoint | Yes | Materials and Methods - 2.2.1 Primary Patient-Disjoint Image-Level Dataset; 2.2.2 Historical Secondary Image-Level Dataset | The primary split is disjoint at infant level. The historical secondary split is image-level and did not enforce patient separation; the manuscript explicitly distinguishes these designs. |
| **21** | Intended test sample size and sample-size determination | No | Materials and Methods - 2.2.1 Primary Patient-Disjoint Image-Level Dataset; Discussion - 4.8 Strengths and Limitations | The achieved cohort size is reported (98 hold-out images from eight infants), but no a priori sample-size calculation or explicit sample-size determination rationale is reported. |
| **22** | Model architecture | Yes | Materials and Methods - 2.5.1 Shared Model Architecture and Training Protocol; 2.5.2 Primary Patient-Disjoint Model Development; 2.5.3 Secondary Random Image-Level Model Development | EfficientNet-B0, DenseNet121, ResNet50/ResNet-18, and MobileNetV3 Large are identified; architecture rationale, classifier heads, and primary/secondary development procedures are reported. |
| **23** | Software libraries/frameworks and versions | No | Materials and Methods - 2.5.1 Shared Model Architecture and Training Protocol | Python random, NumPy, and PyTorch are named in the reproducibility description, but exact software/library version numbers are not reported. |
| **24** | Model parameter initialization | Yes | Materials and Methods - 2.5.1 Shared Model Architecture and Training Protocol | The CNNs were pretrained on ImageNet and fine-tuned after replacement of the original classification head. |
| **25** | Training approach and hyperparameters | Yes | Materials and Methods - 2.5 Model Architectures, Training, and Inference; 2.6 Raw-Fundus Versus Vessel-Overlay Experiment | Data augmentation, optimizer, learning rate, batch size, number of epochs, loss functions, checkpoint criteria, cross-validation strategy, segmentation training details, and vessel-overlay classifier settings are described. |
| **26** | Method for selecting final model(s) | Yes | Materials and Methods - 2.5.1 Shared Model Architecture and Training Protocol; Results - 3.1 Primary Patient-Disjoint Image-Level Performance | Fold-specific checkpoints were selected using validation accuracy. The five architectures were treated as parallel primary models, and hold-out numerical differences were not used to establish statistical superiority or select a definitive architecture. |
| **27** | Ensembling technique | Yes | Materials and Methods - 2.5.2 Primary Patient-Disjoint Model Development; Supplementary Material - Section S1.1.3 | For final evaluation, probabilities from five fold-specific models were averaged to obtain one ensemble probability per image. The reader-comparison model provenance and five-fold ensemble prediction are additionally described in the Supplement. |
| **28** | Performance metrics and clinical relevance | Yes | Materials and Methods - 2.8 Statistical Analysis; Discussion - 4.7 Clinical Operating Thresholds and Potential Clinical Role | Accuracy, sensitivity, specificity, PPV, NPV, F1, MCC, ROC-AUC, PR-AUC, Brier score, confidence intervals, threshold selection, and image-level flagged fraction are defined and clinically contextualized. |
| **29** | Statistical uncertainty/significance | Yes | Materials and Methods - 2.8 Statistical Analysis; Results - 3.1.1 Infant-Cluster Sensitivity Analysis; 3.7 Single-Image Ophthalmologist Reader Study (Table 7) | Wilson intervals are used for selected secondary image-level proportions; cluster-aware uncertainty is explored by 10,000-resample infant-cluster bootstrap and leave-one-infant-out analysis; reader sensitivity/specificity use exact Clopper–Pearson 95% confidence intervals. |
| **30** | Robustness/sensitivity analyses | Yes | Materials and Methods - 2.8 Statistical Analysis; 2.9 Descriptive Within-Infant Prediction Variability; Results - 3.1.1 Infant-Cluster Sensitivity Analysis; 3.1.2 Development-Defined High-Sensitivity Operating Point; 3.2 Descriptive Within-Infant Prediction Variability | Infant-equal weighting, leave-one-infant-out influence analysis, stratified infant-cluster bootstrap, ordered within-infant variability, and cross-architecture variability analyses are reported. |
| **31** | Explainability/interpretability methods | Yes | Results - 3.4 Exploratory Image-Level Explainability and Error Analysis; Supplementary Material - Section S1.2 | Grad-CAM and LIME are reported as exploratory explainability methods. The main manuscript describes LIME retinal-field masking, approximately 30 SLIC superpixels, perturbation-based local surrogate coefficients, and weighted local R²; the Supplement details Grad-CAM generation, fold averaging, target layer, retinal masking, and visualization. Absence of clinician localization masks is explicitly acknowledged. |
| **32** | Internal evaluation | Yes | Results - 3.1 Primary Patient-Disjoint Image-Level Performance; 3.2 Descriptive Within-Infant Prediction Variability; 3.3 Exploratory Secondary Image-Level Architecture Comparison | The primary analysis uses an independent patient-disjoint hold-out from previously unseen infants. The historical secondary held-out image-level analysis is explicitly labelled exploratory because patient separation was not enforced. |
| **33** | External testing | Yes | Discussion - 4.7 Clinical Operating Thresholds and Potential Clinical Role; 4.8 Strengths and Limitations; 5 Conclusion | No external institution/device test cohort was evaluated. The manuscript explicitly reports this limitation and states that larger external, multicentre, device-diverse, and prospective evaluation is required before clinical implementation; the reporting requirement is therefore satisfied transparently without implying external validation. |
| **34** | Clinical-trial registration | N/A | Materials and Methods - 2.1 Ethics Approval; 2.4 Study Design and Analytical Hierarchy | This was a retrospective, non-interventional computational evaluation rather than a prospective clinical trial; trial registration is not applicable. |
| **35** | Numbers included/excluded and participant/image flow diagram | No | Materials and Methods - 2.2.1 Primary Patient-Disjoint Image-Level Dataset; 2.2.2 Historical Secondary Image-Level Dataset; Supplementary Material - Table S1 | Counts for the primary and historical secondary datasets and partitions are reported, but no participant/image flow diagram or complete pre-dataset screening/exclusion log is provided. |
| **36** | Demographic/clinical characteristics by partition | No | Materials and Methods - 2.2 Dataset and Clinical Reference Standard; 2.2.1 Primary Patient-Disjoint Image-Level Dataset; Discussion - 4.8 Strengths and Limitations | Infant/image counts, class distributions, device, and image resolution are reported, but demographic and clinical covariates could not be reliably linked across the complete classification cohort; development-versus-hold-out clinical spectrum balance could not be assessed. |
| **37** | Final performance reporting | Yes | Results - 3.1 Primary Patient-Disjoint Image-Level Performance; 3.2 Descriptive Within-Infant Prediction Variability; 3.3 Exploratory Secondary Image-Level Architecture Comparison; Supplementary Material - Tables S5-S7 | Primary image-level performance is tabulated, with additional cluster-aware analyses, cross-architecture variability, and development-defined high-sensitivity operating-point results. |
| **38** | Precision/uncertainty of diagnostic performance | Yes | Materials and Methods - 2.8 Statistical Analysis; Results - 3.1.1 Infant-Cluster Sensitivity Analysis; 3.7 Single-Image Ophthalmologist Reader Study (Table 7) | The manuscript reports selected Wilson intervals, exploratory cluster-bootstrap AUC percentile intervals, leave-one-infant-out ranges, and exact reader confidence intervals while explicitly avoiding over-interpretation as precise population estimates. |
| **39** | Failure/error analysis | Yes | Results - 3.1 Primary Patient-Disjoint Image-Level Performance; 3.4 Exploratory Image-Level Explainability and Error Analysis; 3.5 Exploratory Secondary Image-Level Probability Reliability | Confusion counts, false-negative burden, selected false-negative cases, Grad-CAM/vessel error visualization, LIME exploratory local explanations, and probability reliability are reported. |
| **40** | Study limitations | Yes | Discussion - 4.8 Strengths and Limitations | Limitations include the small clustered hold-out, unequal images per infant, architecture scope, incomplete metadata, image quality, shortcut learning, historical patient leakage, weak image labels, reader-study size, Grad-CAM spatial limitations, LIME localization-validation limitations, and lack of external validation. |
| **41** | Implications for clinical practice/intended role | Yes | Discussion - 4.7 Clinical Operating Thresholds and Potential Clinical Role; 5 Conclusion | The findings support further investigation of assistive multi-image prioritization/decision-support workflows and explicitly do not support autonomous image-level screening. |
| **42** | Protocol/additional technical details | Yes | Supplementary Material - Sections S1.1-S1.2; Results - 3.4 Exploratory Image-Level Explainability and Error Analysis; Statements - Data Availability Statement | The Supplement provides detailed reader-study and Grad-CAM methods; the main manuscript reports the exploratory LIME procedure; and the public repository is stated to contain preprocessing, training/inference, partitioning, cross-validation, operating-point, variability, and statistical-analysis code. |
| **43** | Software/model/data availability | Yes | Statements - Data Availability Statement | Clinical images cannot be publicly redistributed because of privacy and institutional restrictions; source code and scripts for the reported analyses are publicly available at the stated GitHub repository. |
| **44** | Funding and role of funders | No | Statements - Funding | Funding sources are identified (LERCO and SP2026/032), but the manuscript does not state the role of the funders in study design, data analysis, interpretation, manuscript preparation, or the submission decision. |

Submission note. The checklist is completed as a reporting map for the final uploaded retrospective manuscript and final Supplementary Material. Items are marked “Yes” where the requested information is explicitly reported or where an explicitly reported absence/limitation satisfies the reporting criterion. Remaining “No” and “N/A” responses are intentional and avoid inventing unavailable retrospective information.
