# Supplementary material for: Explainability-informed patient-disjoint evaluation of deep learning for plus disease in retinopathy of prematurity with an ophthalmologist reader study
Source: Front Med (Lausanne). 2026 Sep 30;13:1960317. doi: 10.3389/fmed.2026.1960317 (PMC13661440; doi:10.3389/fmed.2026.1960317)
Supplement: Supplementary file 3 [file Table2.docx]

**STARD-AI Reporting Checklist**

**Explainability-Informed Patient-Disjoint Evaluation of Deep Learning for Plus Disease in Retinopathy of Prematurity with an Ophthalmologist Reader Study**

Completed against the final uploaded main manuscript (43 pages) and final Supplementary Material (9 pages).

Completion note. Every checklist item is answered. “Yes” indicates that the requested reporting element is addressed in the manuscript or Supplement, including transparent reporting that an analysis was not performed or could not be established when that disclosure satisfies the reporting criterion. “No” is reserved for information that is genuinely absent or not recoverable, and “N/A” for items that are not applicable. No unavailable information has been inferred or reconstructed.

**Guideline:** Sounderajah V, Guni A, Liu X, et al. The STARD-AI reporting guideline for diagnostic accuracy studies using artificial intelligence. Nature Medicine. 2025;31:3283–3289. DOI: 10.1038/s41591-025-03953-8.

| **Item** | **Reporting criterion** | **Status** | **Section / locator reference** | **Completed response / explanation** |
| --- | --- | --- | --- | --- |
| **1** | Identify the study as evaluating an AI index test and include an accuracy measure in title/abstract | Yes | Abstract | The title identifies deep learning, and the abstract reports ROC-AUC, sensitivity, specificity, and patient-disjoint evaluation. |
| **2** | Structured abstract | Yes | Abstract | The abstract is structured into Background, Methods, Results, and Conclusions and summarizes the diagnostic-accuracy evaluation. |
| **3** | Scientific/clinical background, intended use and clinical workflow | Yes | Introduction; Discussion - 4.7 Clinical Operating Thresholds and Potential Clinical Role | Clinical ROP/plus-disease assessment, multi-image review, AI motivation, limitations of single frames, and a potential assistive workflow are described. |
| **4** | Specific objectives/hypotheses | Yes | Introduction - Study objectives | Six explicit objectives are provided. |
| **5** | Prospective versus retrospective design | Yes | Materials and Methods - 2.1 Ethics Approval; 2.4 Study Design and Analytical Hierarchy | The study is explicitly retrospective. |
| **6** | Ethics approval | Yes | Materials and Methods - 2.1 Ethics Approval; Statements - Ethics Statement | Ethics Committee of University Hospital Ostrava approval no. 544/2018, de-identification, institutional governance, and Declaration of Helsinki compliance are reported. A separate consent/waiver statement is not included in the manuscript. |
| **7** | Eligibility criteria at participant and data levels | No | Materials and Methods - 2.2.1 Primary Patient-Disjoint Image-Level Dataset; 2.2.2 Historical Secondary Image-Level Dataset; 2.5 Model Architectures, Training, and Inference | The analyzed cohorts and absence of post-hoc subjective quality exclusion are described, but explicit participant-level and image-level eligibility/exclusion criteria are not fully reported. |
| **8** | Basis for identifying potentially eligible participants/data | No | Materials and Methods - 2.2 Dataset and Clinical Reference Standard; 2.2.1 Primary Patient-Disjoint Image-Level Dataset; 2.2.2 Historical Secondary Image-Level Dataset | The retrospective Phoenix ICON source collections are described, but the method used to identify all potentially eligible infants/images from the parent archive is not reported. |
| **9** | Setting, location, and relevant dates | Yes | Materials and Methods - 2.1 Ethics Approval; 2.2 Dataset and Clinical Reference Standard | The study setting (University Hospital Ostrava), imaging device (Phoenix ICON), and retrospective context are reported. Exact examination/acquisition dates could not be consistently linked across the classification cohort and this limitation is explicitly reported; no dates were retrospectively inferred. |
| **10** | Whether the participant/data series was consecutive, random, or convenience based | No | Materials and Methods - 2.2 Dataset and Clinical Reference Standard; 2.2.1 Primary Patient-Disjoint Image-Level Dataset; 2.2.2 Historical Secondary Image-Level Dataset | The primary source-series sampling type is not stated. The historical secondary partition used stratified random image-level sampling, but that does not define how the source cohort itself was assembled. |
| **11** | Data source and collection method | Yes | Materials and Methods - 2.2 Dataset and Clinical Reference Standard; 2.2.1 Primary Patient-Disjoint Image-Level Dataset; 2.2.2 Historical Secondary Image-Level Dataset | Institutional retrospective Phoenix ICON image collections, the primary patient-disjoint dataset, and the historical secondary dataset are described. |
| **12** | Dataset annotation: annotators, method, and clinical context | Yes | Materials and Methods - 2.2 Dataset and Clinical Reference Standard | Binary clinical labels were assigned by one of two experienced ROP ophthalmologists (J.T. and K.M.) after review of the complete available fundus-image set for the corresponding clinical case; each infant was assessed by one ophthalmologist. |
| **13** | Capture device(s) and software/framework versions | No | Materials and Methods - 2.2 Dataset and Clinical Reference Standard; 2.5.1 Shared Model Architecture and Training Protocol | Phoenix ICON and software/framework names are reported, but exact software/library version numbers are not reported. |
| **14** | Acquisition protocols and preprocessing | Yes | Materials and Methods - 2.2 Dataset and Clinical Reference Standard; 2.5 Model Architectures, Training, and Inference | Image device/resolution plus cropping, resizing, ImageNet normalization, augmentation, and absence of validation/test-time augmentation are reported. |
| **15a** | Index test described with sufficient technical detail for replication | Yes | Materials and Methods - 2.5.1 Shared Model Architecture and Training Protocol; 2.5.2 Primary Patient-Disjoint Model Development; 2.5.3 Secondary Random Image-Level Model Development; 2.6 Raw-Fundus Versus Vessel-Overlay Experiment | Architecture panel, classifier heads, training settings, cross-validation, ensemble inference, and the vessel-overlay experiment are described in technical detail. |
| **15b** | Index-test development, training, validation, and testing chronology | Yes | Materials and Methods - 2.2.1 Primary Patient-Disjoint Image-Level Dataset; 2.2.2 Historical Secondary Image-Level Dataset; 2.5.1 Shared Model Architecture and Training Protocol; 2.5.2 Primary Patient-Disjoint Model Development; 2.5.3 Secondary Random Image-Level Model Development; 2.8 Statistical Analysis | Primary and secondary partitioning, cross-validation, checkpoint selection, hold-out exclusion, ensemble evaluation, and development-only sensitivity-oriented threshold selection are reported. |
| **15c** | Index-test cut-offs/result categories and how selected | Yes | Materials and Methods - 2.5.2 Primary Patient-Disjoint Model Development; 2.5.3 Secondary Random Image-Level Model Development; 2.8 Statistical Analysis | The fixed threshold of 0.5 and the separate development-defined high-sensitivity operating point are described and distinguished; no hold-out optimization was used for the latter. |
| **15d** | Intended end user and required expertise | Yes | Discussion - 4.3 Within-Infant Prediction Variability and Descriptive Aggregation; 4.7 Clinical Operating Thresholds and Potential Clinical Role | The proposed future workflow presents examination-level outputs and representative images for ophthalmologist review and is explicitly framed as assistive multi-image decision support rather than autonomous screening. |
| **16a** | Reference standard described | Yes | Materials and Methods - 2.2 Dataset and Clinical Reference Standard | The clinical image-set/examination-derived binary plus/non-plus reference standard is clearly described. |
| **16b** | Rationale for choice of reference standard | Yes | Materials and Methods - 2.2 Dataset and Clinical Reference Standard; Discussion - 4.2 Clinical Reference Standard and Unit of Analysis | The reference reflects the available clinical image-set assessment rather than independent grading of every photograph. The manuscript explains why this distinction matters and treats the inherited image labels as weak supervision. |
| **16c** | Reference-standard positivity/categories | Yes | Introduction; Materials and Methods - 2.2 Dataset and Clinical Reference Standard | Plus disease is clinically defined; the classification cohort uses binary plus/non-plus labels. Pre-plus was not encoded as a separate reference category and was not retrospectively assigned. |
| **17a** | Reference information available to the index-test performer/model during evaluation | Yes | Materials and Methods - 2.5.2 Primary Patient-Disjoint Model Development; 2.5.3 Secondary Random Image-Level Model Development; 2.8 Statistical Analysis | The independent primary hold-out was excluded from model fitting, validation, checkpoint selection, and threshold selection. Hold-out labels were used for final evaluation rather than model optimization. |
| **17b** | Index-test results available to the reference-standard assessor | Yes | Materials and Methods - 2.2 Dataset and Clinical Reference Standard; 2.2.2 Historical Secondary Image-Level Dataset | The reference labels are described as pre-existing clinical labels assigned by ophthalmologists from the clinical image sets. Model predictions are not described as part of the original reference-standard construction. |
| **18** | Methods for diagnostic-accuracy estimates and comparisons | Yes | Materials and Methods - 2.8 Statistical Analysis | Threshold-dependent formulas, MCC, ROC-AUC, PR-AUC, Brier score, Wilson intervals, cluster-aware analyses, and reader-analysis statistics are defined. |
| **19** | Handling of indeterminate index-test/reference-standard results | N/A | Materials and Methods - 2.2 Dataset and Clinical Reference Standard; 2.8 Statistical Analysis | The analyzed reference labels and model classification outputs are binary; no indeterminate index-test or reference category was used. |
| **20** | Handling of missing index-test/reference-standard data | Yes | Materials and Methods - 2.2 Dataset and Clinical Reference Standard; Results - 3.1 Primary Patient-Disjoint Image-Level Performance | The reported classification analyses use defined binary reference labels and model outputs for the analyzed images. Missing ancillary clinical/acquisition covariates were not retrospectively inferred or imputed. |
| **21** | Variability analyses and identification of exploratory analyses | Yes | Materials and Methods - 2.8 Statistical Analysis; 2.9 Descriptive Within-Infant Prediction Variability | Infant-equal weighting, leave-one-infant-out influence, infant-cluster bootstrap, ordered within-infant variability, and cross-architecture analyses are explicitly described as additional/exploratory where appropriate. |
| **22** | Intended sample size and how it was determined | No | Materials and Methods - 2.2.1 Primary Patient-Disjoint Image-Level Dataset; Discussion - 4.8 Strengths and Limitations | The achieved test cohort size is reported, but no a priori sample-size calculation or explicit sample-size determination rationale is provided. |
| **23** | Error analysis and assessment of algorithmic bias/fairness | Yes | Results - 3.4 Exploratory Image-Level Explainability and Error Analysis; Discussion - 4.8 Strengths and Limitations | Performance error analysis was undertaken and reported, including false-negative examples, Grad-CAM findings, complementary LIME local explanations, and consideration of possible shortcut features. Formal subgroup-based algorithmic bias/fairness analysis was not undertaken because the linked demographic and clinical variables required for reliable stratification were incomplete; this limitation is explicitly reported. |
| **24** | Participant/data flow diagram | No | Materials and Methods - 2.2.1 Primary Patient-Disjoint Image-Level Dataset; 2.2.2 Historical Secondary Image-Level Dataset; Supplementary Material - Table S1 | Dataset and partition counts are reported, but no participant/data flow diagram or complete pre-dataset screening/exclusion pathway is provided. |
| **25** | Baseline demographic, clinical, and technical characteristics by split | No | Materials and Methods - 2.2 Dataset and Clinical Reference Standard; 2.2.1 Primary Patient-Disjoint Image-Level Dataset; 2.2.2 Historical Secondary Image-Level Dataset; Discussion - 4.8 Strengths and Limitations | Technical acquisition information, cohort counts, and class distributions are provided, but demographic/clinical baseline characteristics could not be reliably linked across the complete classification cohort. |
| **26a** | Distribution of disease severity in target-positive participants/data | No | Materials and Methods - 2.2 Dataset and Clinical Reference Standard | The classification reference is binary plus/non-plus. Separate pre-plus, standardized ROP stage/zone, and image-level severity distributions are unavailable across the complete cohort. |
| **26b** | Alternative diagnoses in target-negative participants/data | No | Materials and Methods - 2.2 Dataset and Clinical Reference Standard | Alternative diagnoses within the non-plus reference group are not reported as standardized linkable variables for the classification cohort and were not retrospectively inferred. |
| **27** | Time interval/interventions between index test and reference standard | N/A | Materials and Methods - 2.2 Dataset and Clinical Reference Standard; 2.2.1 Primary Patient-Disjoint Image-Level Dataset; 2.2.2 Historical Secondary Image-Level Dataset | This is a retrospective AI analysis of stored fundus images with existing clinical reference labels; no contemporaneous diagnostic intervention interval between a prospective index test and reference standard applies. |
| **28** | Representativeness of the evaluation set for the intended-use population | Yes | Materials and Methods - 2.2.1 Primary Patient-Disjoint Image-Level Dataset; Discussion - 4.8 Strengths and Limitations | Representativeness is explicitly addressed. The primary hold-out contains only eight infants from a single institutional/device context, and incomplete clinical covariates prevented confirmation that its clinical spectrum reflects the intended-use population. The manuscript therefore restricts generalizability and calls for larger external, multicentre, device-diverse prospective evaluation. |
| **29** | Differences in an external evaluation dataset | N/A | Discussion - 4.8 Strengths and Limitations; 5 Conclusion | No external institutional/device evaluation dataset was used; therefore comparison of development versus external-evaluation characteristics is not applicable. |
| **30** | Cross-tabulation/distribution of index-test results against the reference standard | Yes | Results - 3.1 Primary Patient-Disjoint Image-Level Performance; 3.3 Exploratory Secondary Image-Level Architecture Comparison; 3.7 Single-Image Ophthalmologist Reader Study (Table 7) | Primary DenseNet121 and secondary ResNet-18 confusion counts and reader-reference results are reported, with tabulated performance measures. |
| **31** | Diagnostic-accuracy estimates with measures of precision | Yes | Materials and Methods - 2.8 Statistical Analysis; Results - 3.1.1 Infant-Cluster Sensitivity Analysis; 3.7 Single-Image Ophthalmologist Reader Study (Table 7) | Cluster-bootstrap AUC percentile intervals, leave-one-infant-out ranges, selected Wilson confidence intervals, and exact Clopper–Pearson reader confidence intervals are reported. The manuscript explicitly states that the small number of infant clusters limits population-level precision. |
| **32** | Adverse events from the index test or reference standard | N/A | Materials and Methods - 2.1 Ethics Approval | The study is a retrospective software/image-analysis evaluation with no interventional index test performed on participants as part of this study; a test-related adverse-event analysis is not applicable. |
| **33** | Limitations, including bias, uncertainty, and generalizability | Yes | Discussion - 4.8 Strengths and Limitations | The limitations section addresses small clustered data, unequal images per infant, architecture scope, missing metadata, image quality, shortcut learning, historical leakage, weak labels, reader-study size, Grad-CAM spatial limitations, LIME localization-validation limitations, and lack of external validation. |
| **34** | Implications for practice and intended clinical role | Yes | Discussion - 4.3 Within-Infant Prediction Variability and Descriptive Aggregation; 4.7 Clinical Operating Thresholds and Potential Clinical Role; 5 Conclusion | The system is framed as a research component of assistive multi-image prioritization/decision support and not as autonomous screening; prospective validation is required. |
| **35** | Ethical considerations and fairness | Yes | Materials and Methods - 2.1 Ethics Approval; Discussion - 4.8 Strengths and Limitations; Statements - Ethics Statement | Ethics approval, de-identification, data-governance requirements, and Declaration of Helsinki compliance are reported. Fairness/equity cannot be formally established because demographic and clinical subgroup variables were incompletely linked; the manuscript explicitly reports the inability to perform reliable subgroup analyses together with potential shortcut-learning and generalizability concerns. |
| **36** | Registration | N/A | Materials and Methods - 2.1 Ethics Approval; 2.4 Study Design and Analytical Hierarchy | This retrospective, non-interventional computational study was not presented as a prospectively registered diagnostic trial; registration is not applicable. |
| **37** | Access to the full study protocol | N/A | Statements - Data Availability Statement; Supplementary Material - Sections S1.1-S1.2 | No separate prospectively registered full study protocol was used for this retrospective, non-interventional computational analysis. Detailed methods, supplementary procedures, and public analysis/training code are provided; therefore a separate protocol-access location is not applicable. |
| **38** | Funding source and role of the funder | No | Statements - Funding | Funding sources are reported, but the role of the funders in study design, data analysis, interpretation, manuscript preparation, or the submission decision is not stated. |
| **39** | Commercial interests | Yes | Statements - Conflict of Interest | The authors declare absence of commercial or financial relationships that could be construed as a potential conflict of interest. |
| **40a** | Dataset/code availability and restrictions on reuse | Yes | Statements - Data Availability Statement | Clinical retinal images are not publicly redistributable because of privacy/data-governance restrictions. Source code and analysis scripts are publicly available at the reported GitHub repository. |
| **40b** | Stored/auditable model outputs available for evaluation | No | Statements - Data Availability Statement | The manuscript states that scripts reproduce analyses when used with the corresponding clinical data and derived model outputs, but it does not state that per-image prediction outputs or audit logs are publicly available or independently auditable. |

Submission note. The checklist is completed as a reporting map for the final uploaded retrospective manuscript and final Supplementary Material. Items are marked “Yes” where the requested information is explicitly reported or where an explicitly reported absence/limitation satisfies the reporting criterion. Remaining “No” and “N/A” responses are intentional and avoid inventing unavailable retrospective information.
